# ‘Glocal’ Robustness Analysis and Model Discrimination for Circadian Oscillators

**DOI:** 10.1371/journal.pcbi.1000534

**Published:** 2009-10-16

**Authors:** Marc Hafner, Heinz Koeppl, Martin Hasler, Andreas Wagner

**Affiliations:** 1 School of Computer and Communication Sciences, Ecole Polytechnique Fédérale de Lausanne, Lausanne, Switzerland; 2 Department of Biochemistry, University of Zurich, Zurich, Switzerland; 3 Swiss Institute of Bioinformatics, Lausanne, Switzerland; 4 Plectix Biosystems, Somerville, Massachussetts, United States of America; 5 The Santa Fe Institute, Santa Fe, New Mexico, United States of America; 6 Department of Biology, University of New Mexico, Albuquerque, New Mexico, United States of America; University of Virginia, United States of America

## Abstract

To characterize the behavior and robustness of cellular circuits with many unknown parameters is a major challenge for systems biology. Its difficulty rises exponentially with the number of circuit components. We here propose a novel analysis method to meet this challenge. Our method identifies the region of a high-dimensional parameter space where a circuit displays an experimentally observed behavior. It does so via a Monte Carlo approach guided by principal component analysis, in order to allow efficient sampling of this space. This ‘global’ analysis is then supplemented by a ‘local’ analysis, in which circuit robustness is determined for each of the thousands of parameter sets sampled in the global analysis. We apply this method to two prominent, recent models of the cyanobacterial circadian oscillator, an autocatalytic model, and a model centered on consecutive phosphorylation at two sites of the KaiC protein, a key circadian regulator. For these models, we find that the two-sites architecture is much more robust than the autocatalytic one, both globally and locally, based on five different quantifiers of robustness, including robustness to parameter perturbations and to molecular noise. Our ‘glocal’ combination of global and local analyses can also identify key causes of high or low robustness. In doing so, our approach helps to unravel the architectural origin of robust circuit behavior. Complementarily, identifying fragile aspects of system behavior can aid in designing perturbation experiments that may discriminate between competing mechanisms and different parameter sets.

## Author Summary

Robustness is an intrinsic property of many biological systems. To quantify the robustness of a model that represents such a system, two approaches exist: global methods assess the volume in parameter space that is compliant with the proper functioning of the system; and local methods, in contrast, study the model for a given parameter set and determine its robustness. Local methods are fundamentally biased due to the *a priori* choice of a particular parameter set. Our ‘glocal’ analysis combines the two complementary approaches and provides an objective measure of robustness. We apply this method to two prominent, recent models of the cyanobacterial circadian oscillator. Our results allow discriminating the two models based on this analysis: both global and local measures of robustness favor one of the two models. The ‘glocal’ method also identifies key factors that influence robustness. For instance, we find that in both models the most fragile reactions are the ones that affect the concentration of the feedback component.

## Introduction

Biologists' qualitative reasoning about outcomes of experiments show inherent limitations. Mathematical models of cellular processes, such as signaling, cell-cycle regulation, or circadian rhythmicity [1],[2] can compensate for these limitations. Such models are often systems of ordinary differential equations, whose state variables represent the molecules that take part in a process. The interactions between molecules are encapsulated in the differential equations themselves, where multiple biochemical parameters determine rates at which molecules are synthesized or degraded, at which they associate, dissociate, or are transformed into other molecules. Although some data on a cellular process often exists to inform such models, substantial uncertainty often remains about which molecular interactions occur in it, and about values of the parameters governing these interactions [3].

When given two models for the same cellular process, which one is better in the face of such uncertainty about model structure and parameters? Traditionally, this question has often been approached by model calibration [4]. Here, a model is judged superior if there exist parameters (in its usually high-dimensional *parameter space*) that allow the model to mimic biologically observed behavior more closely than other models. This approach fails in the common situation where parameters are underdetermined by model behavior and thus many parameter sets exist that match the behavior equally well [5]. That this deficiency is particularly pronounced for models of cellular processes was shown in [6].

A system is called robust to a specific class of perturbations if it can maintain its function or structure under these perturbations [7]. Such perturbations include changes in biochemical parameters (e.g. temperature [8] and other environmental changes), molecular noise [9]–[12], changes of molecular concentrations, as well as mutations [13],[14]. Many properties of biochemical systems show some robustness to such perturbations [15]–[21]. These observations raise the possibility that robustness itself could be used to discriminate between models [15],[16]. In the absence of other criteria, a model would be judged superior if it is more robust than other models to some class of perturbations [19]. This notion forms the cornerstone of our contribution.

Conventional methods used in robustness analysis can be subdivided into global and local methods. Global methods characterize properties of a model's parameter space, such as the size or volume that generate a behavior of interest [22],[23], or a parameter's bifurcation diagram [24]–[26]. Such a diagram characterizes how qualitative model properties, such as stability of steady-states, change as model parameters are varied [27]. The structure of a bifurcation diagram can be influenced by variation in parameters that are not considered, which limits this approach. Finally, multivariate continuation methods [28] do not show a strong advantage over unrestricted sampling for high-dimensional systems, as they reduce the sampling space only by one dimension.

In contrast to global methods, local methods analyze how perturbations affect model behavior for one specific set of parameters. Their main limitation is precisely this: they may not reflect model behavior under all possible parameters sets. Most robustness analyses in the literature are local. Examples include sensitivity analysis [29], which studies the effect of perturbations for a given parameter set on model behavior, and its application to circadian oscillators [17],[18],[29]. These methods are usually based on the linearization of a system and therefore hold for variations of only a few percent of the parameter values. Other work uses stochastic simulations to estimate the robustness of a system to molecular noise [11],[12]. Efforts to extend a local analysis to systematic parameter variations in more than one or two dimensions [25],[26] are often limited by computational cost.

We here propose a novel ‘glocal’ method for analysis and quantification of robustness that combines a global with a local approach. Understanding the origin of robustness and fragility may inform new experiments that can best discriminate between competing hypothetical mechanisms or models. Briefly, the global approach aims at estimating the volume in parameter space occupied by parameters for which a model yields a biologically observed behavior. Because such a search becomes very challenging in high-dimensional parameter spaces, we guide this search through an iterative procedure that involves principal component analysis (PCA) [30]. The second, local aspect of our method evaluates the robustness of model behavior – for each of the previously generated parameter sets – to five different kinds of perturbations, including concentration perturbations and molecular noise. Conceptually our method is different from parameter fitting in the sense that it provides the parameter region where the model is consistent with experimental observations instead of a single parameter set.

To illustrate the application of our method, we focus on two recent models of the cyanobacterial circadian oscillator [31],[32]. Circadian oscillators drive activity patterns of a 24 hour period in many animals, most plants [33], and some bacteria [34]. In cyanobacteria, the purpose of this oscillator is to regulate gene expression, mainly in order to alternate between the exclusive processes of nitrogen fixation and photosynthesis according to light availability [34]. Experiments with mutants have shown that cyanobacteria with a too short- or long-period are eliminated under selection pressure against wild-type organisms synchronized with the 24-hours light/dark cycle [35]. The cyanobacterial oscillator has been reconstituted in vitro [36], and is one of the simplest known in any organism [34]. It involves three main proteins called KaiA, KaiB and KaiC. When mixed with ATP, reaction buffer and appropriate concentrations of KaiA and KaiB, KaiC continuously oscillates between a low phosphorylated state and a high one [36]. KaiA and KaiB modulate the phosphorylation status of KaiC. Specifically, KaiA catalyzes KaiC phosphorylation and also seems to inhibit its dephosphorylation. KaiB antagonizes the action of KaiA when KaiC is highly phosphorylated [37]. Highly phosphorylated KaiC is likely to be the readout component because it can bind DNA [38] and thus regulate the expression of other genes. In vivo, additional proteins interact with the three core proteins to entrain the cycle and communicate the output signal to the cell. We here focus on models that involve the three core proteins, because these are necessary and sufficient for autonomous oscillations.

We chose this study system for several reasons. First, it is an area of very active recent model development, [31],[32],[37],[39],[40], driven by recent insights into the molecular mechanisms of the oscillator [36]. Second, the behavior or function of circadian oscillators is well-characterized: an ample oscillation with a period of approximately 24 hours [34], and low sensitivity to non-periodic environmental perturbations. Third, in vitro and in vivo experiments show that the cyanobacterial circadian clock is robust to many perturbations [41],[42]. Fourth, good estimates for the in vivo abundance of all involved proteins and of the cell volume for the cyanobacteria are available. Finally, being posttranslational, the clock shares many features with signal transduction pathways, an important field of application for robustness analysis [43],[44]. In order to relate our work to previous robustness studies on transcriptional circadian oscillators [1],[2],[45],[46] we also characterize a prototypical such oscillator in the supplementary material (see Text S1, section C).

## Results

### ‘Glocal’ Robustness

A model's behavior is determined by some number 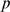 of parameters, i.e., the *parameter vector* 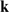. Any robustness analysis needs to quantitatively characterize the system's function that is maintained under perturbations. We do this through a collection of *systemic properties* 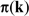 that are required to assume values within predetermined intervals. In our application 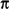 comprises the period 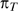 and amplitude 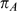 of the circadian oscillation of phosphorylated KaiC. We say the oscillator with parameter vector 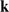 maintains its function and *preserves* 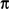 if 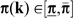, where we chose these bounds [35] to be 10% below and above published values [31],[32]. In the following passages, however, we refer to some general and hypothetical vector of properties 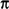 to emphasize the generality of our approach.

**Figure 1:**
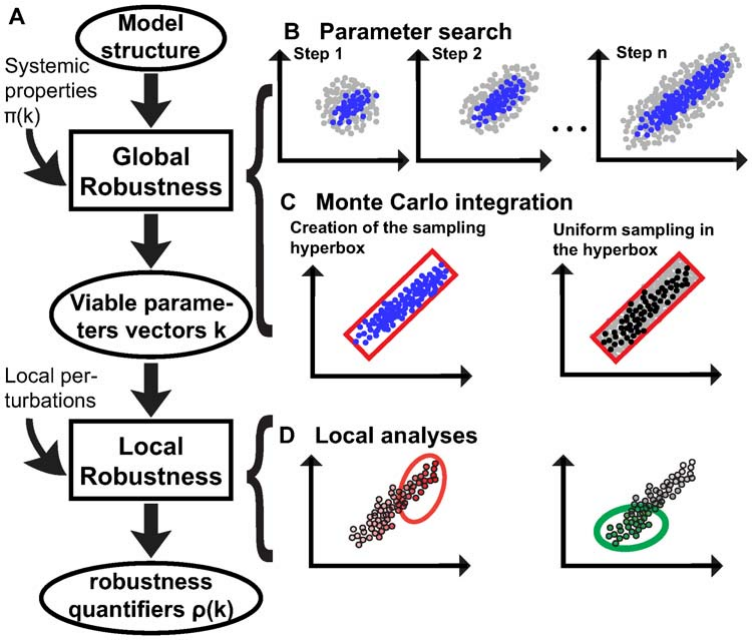
Glocal robustness analysis flow for a hypothetical two dimensional parameter space. (A) A model structure and systemic properties serve as inputs for the global step of the analysis. This global analysis is composed of (B) and (C) and yields viable parameter vectors 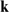 for the model in addition to the normalized viable volume 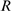. Different local perturbations are applied to these parameter vectors (D) in order to quantify their local robustness 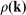. (B) Monte Carlo sampling to define viable parameter ranges. The first sampling step uses Gaussian random sampling with independently and identically distributed random variables. Some of the tested parameter vectors (gray circles) are viable (blue circles). For subsequent iterative steps, sampling occurs according to the covariance matrix of viable parameters estimated in previous steps. (C) Monte Carlo integration. To estimate the volume in which viable parameter sets occur, we define a hyperbox (red rectangle in left panel) that contains all the viable parameters of the last iteration. We then sample uniformly parameter vectors from this box (right panel, gray circles) and estimate the fraction of viable parameter vectors (right panel, black circles). (D) Local analyses are performed on all viable parameter vectors and help identify correlations between parameter vectors or their components, and robustness values (color intensity) to provide regions of high robustness in the parameters space; two different local robustness quantifiers (left-red, and right-green).

The first step of our approach involves the sampling of a large set 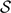 of vectors 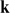 that span several orders of magnitude for each component. Only a subset 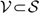 will generally preserve 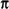. We call such parameter vectors *viable*. We sample according to an iterative scheme, where in each step the sampling distribution is adjusted based on a PCA of the viable set of the previous step (Figure 1A). After a Monte Carlo integration (Figure 1B), the volume occupied by the set 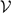 provides a first, crude characterization of a model's robustness and can aid in model discrimination by proper normalization. Unless otherwise mentioned, all calculations and observations below are made in the decadic logarithmic domain, because of the broad ranges of parameter values we explore.

The next step of our approach takes advantage of *all* previously identified viable parameter vectors in order to carry out a local robustness analysis. (Figure 1C). This is done by defining a vector of robustness quantifiers 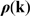 for each 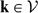. Specifically, we use five complementary quantifiers to assess the robustness of model properties 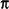 to particular kinds of perturbations. We normalize the local robustness quantifiers to range from zero (minimal robustness) to one (maximal robustness). Because the set 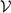 consists of a finite number of sampled parameter vectors, we use statistical tests to assess the results of our analyses. In *Methods* we provide details on the iterative sampling scheme, the volume occupied by 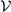, and the five local robustness quantifiers.

### Two oscillator models

We now apply our approach to two recently proposed mathematical models of the cyanobacterial circadian oscillator. As briefly discussed above, this oscillator involves three core proteins, KaiA, KaiB, and KaiC, which form complexes with one another (denoted as KaiAB, KaiABC, etc.).

**Figure 2:**
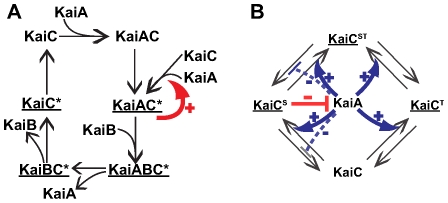
Two models of the cyanobacterial circadian cycle. (A) Autocatalytic model from Mehra et al. [31]. ‘C*’ stands for phosphorylated KaiC. The cycle proceeds clockwise, starting from the upper left. The sum of concentrations of the KaiC*-containing complexes (underlined) form the output of the model. The red arrow denotes the autocatalytic effect of KaiAC* on its synthesis. (B) Two phosphorylation sites model from Rust et al. [32]. There are three possible phosphorylated states for KaiC: 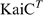, 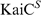 and 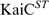. The sum of concentrations of phosphorylated KaiC molecules (underlined) is the output of the system. KaiA catalyzes phosphorylation reactions (solid blue arrows) and inhibits some dephosphorylation reactions (dashed blue bars). 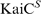 (complexed with KaiB, not explicitly modeled) inhibits the action of KaiA (red bar).

The first model [31] (Figure 2A, see Text S1, section A.4, for equations) involves complex formation of KaiC with the other proteins, as well as cyclic phosphorylation and desphosphorylation of KaiC. In this model, KaiA first binds to KaiC (top reaction of Figure 2A). The resulting complex KaiAC catalyzes the phosphorylation of KaiC forming KaiAC*. A central element of this model is that KaiAC* then exerts a positive feedback on its own formation (red arrow in Figure 2A). In a subsequent step, KaiB binds to the complex KaiAC* and inhibits this autocatalysis. To complete the cycle, KaiA is released, followed by KaiB, and KaiC* is dephosphorylated. We will refer to this model as the *autocatalytic* model.

The second model [32] (Figure 2B, equations in Text S1, section A.5) takes into account two sites S and T of phosphorylation for KaiC [47], resulting in three possible phosphorylated states: 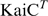, 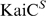 and 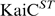. KaiA catalyzes the phosphorylation of KaiC, 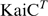 and 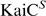 and inhibits the dephosphorylation of 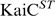 and 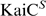. These actions of KaiA are inhibited by 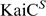 (red bar in Figure 2B). Although 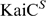 exerts its effects on KaiA jointly with KaiB [48], KaiB does not appear in the equations, because it is assumed to be at saturation level in this model. We will refer to this model as the *two (phosphorylation) sites* model.

Both models capture important empirical observations about the cyanobacterial circadian cycle: phosphorylation of KaiC with the help of KaiA [47], inhibition of this effect by KaiB when bound to phosphorylated KaiC [47]–[49], and finally dephosphorylation to complete the cycle [47]. However, the models are also fundamentally different in some key assumptions about the underlying mechanism. Because of these dramatic differences, biochemical data will play a decisive role in model discrimination. The robustness analysis we carry out is a first step towards such validation.

### The two-sites model shows greater global robustness

In applying our global approach to both models, we sampled parameter vectors covering an enormous range of six orders of magnitude for each parameter, centered on published [31],[32] parameter values for both models (see *Methods* and Text S1, sections A.4 and A.5, for parameter values). We carried out our procedure for ten PCA iterations and used the viable parameters of the last four iterations to define the hyperbox for the Monte Carlo integration.

**Figure 3:**
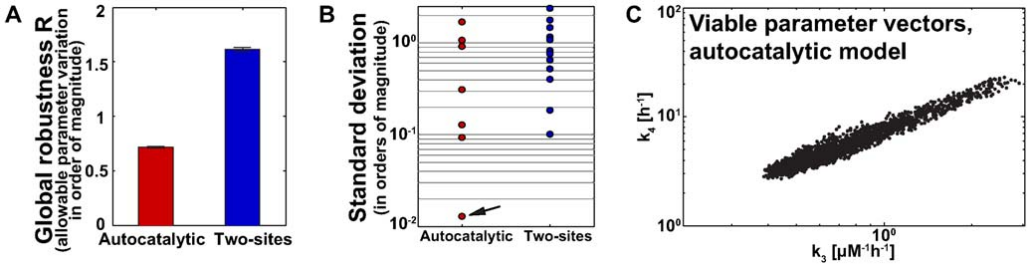
Results of the global robustness analyses for both models. (A) The two-sites model (right) has significantly greater nomalized viable volume than the autocatalytic model (left). Error bars (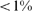) correspond to standard deviations over five independent estimates. (B) Standard deviations along the principal axes of viable parameters for the autocatalytic model and the two-sites model. Note the logarithmic scale. The autocatalytic model has a strongly constrained axis (arrow); amounts of variation along the other axes are overall smaller for the autocatalytic model. (C) Projection of the viable vectors of the autocatalytic model after the MC integration on the plane 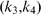. These two parameters are strongly correlated resulting in the lowest standard deviation for the autocatalytic model (B).

Figure 3A shows the (normalized) viable volumes 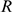 for the two models. These volumes can be interpreted as the average allowable variation per parameter that leaves the circadian oscillations intact. The two-sites model is vastly more robust than the autocatalytic model. Specifically, the value 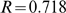 for the autocatalytic model means that the parameters can vary over 0.7 orders of magnitude, or 5.2-fold. For the two-sites model, the value of 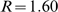 is more than twice that, correspond to a 39-fold allowable variation. The values shown are based on at least 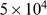 parameter vectors and have sampling errors of less than one percent (see *Methods* for details). We also note that the estimated viable parameter volumes were highly reproducible among five independent applications of the iterative procedure. For example, the mean values of 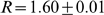 (two-sites model) and 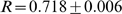 (autocatalytic model) have a coefficient of variation below one percent over these five iterations, which shows that the PCA-guided sampling approach gives highly reproducible results.

What is responsible for the lower robustness of the autocatalytic model? One possibility is that strong associations exist between individual parameters in viable parameter sets, such that some parameters cannot vary independently from others. Such associations, if present, may also provide mechanistic insights into complex, high-dimensional circuits. Figure 3B shows the standard deviations of viable parameters along the principal axes of both models. With one exception, the amount of variation along most principal component axes is similar for both models. The exception (indicated by the arrow in the Figure 3B) is the lowest PCA axis for the autocatalytic model.

The high constraint on variation in this axis is caused by a strong positive correlation between the rate for the autocatalytic reaction, parameter 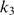, and the rate for the formation of the complex KaiABC*, 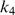 (Figure 3C). This axis deviates by merely 13 degrees from the vector 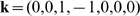 defined by these parameters. Parameters 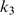 and 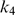 are highly correlated (Pearson's 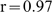, significance of all statistical tests are summarized in Table 1). This strong association contributes to the lack of global robustness we observe in the autocatalytic model. It means that a perturbation of parameter 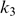 that would not be followed by a corresponding perturbation in parameter 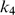 would prevent the model to preserve properties 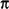 of interest. When we examine the structure of the equations for the autocatalytic model (Figure 2A), we find that the mechanistic cause for this association lies in the dynamics of KaiAC*: on the one hand, if 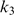 is too large, the concentration of KaiAC* increases too fast and the autocatalytic effect is too strong; on the other hand, if 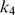 is too large, the concentration of KaiAC* is too low and the autocatalytic effect is too weak. The parameters 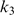 and 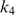 need to be delicately balanced to have the correct concentration of KaiAC* resulting in the appropriate feedback strength.

**Table 1:** Statistical tests and their significance used to assess model discrimination and correlations.

Null hypothesis	Test type	r-value	p-value	n
Parameters 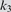 and 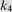 are correlated in the autocatalytic model	Pearson's	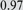	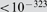	1828
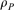 is larger for two-sites model	Wilcoxon rank		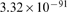	
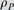 correlated with 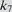 for autocatalytic model	Spearman's	−0.638	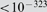	1828
robustness to temperature changes is larger than 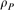 for autocatalytic model	Wilcoxon rank sum		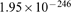	
robustness to temperature changes is larger than 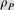 for two-sites model	Wilcoxon rank sum		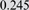	
robustness to temperature changes is larger for two-sites model	Wilcoxon rank sum		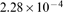	
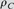 is larger for two-sites model	Wilcoxon rank		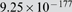	
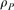 correlated with 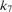 for autocatalytic model	Spearman's	−0.718	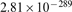	1828
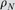 is larger for two-sites model	Wilcoxon rank		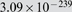	
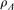 is larger for two-sites model	Wilcoxon rank		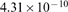	
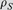 is larger for two-sites model	Wilcoxon rank		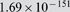	
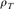 is larger for two-sites model	Wilcoxon rank		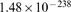	
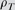 is correlated with the distance from the parameter with the highest 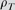 for autocatalytic model	Spearman's	−0.355	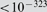	1828
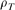 is correlated with the distance from the parameter with the highest 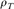 for two-sites model	Spearman's	−0.196	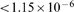	604

To assess whether this strong association is responsible for the smaller global robustness of the autocatalytic model, we collapsed the highly correlated parameters 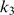 and 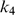 into one. That is, we assumed that 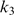 and 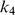 are linearly dependent and can be considered as one single parameter. The reduced model with only six parameters yields a global robustness estimate of 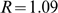. This corresponds to an allowable 12-fold average variation of each parameter, and accounts partially for the lower robustness of the autocatalytic model.

A remaining question is whether the viable region of parameter space forms a connected set. Such connectedness would facilitate the evolution of oscillators with high robustness through gradual changes of individual parameters. Although this question cannot be answered rigorously by our sampling approach we show that this is probably the case for both models (Text S1, section B.1, and Figure S1).

### The two-sites model shows greater overall local robustness

**Figure 4:**
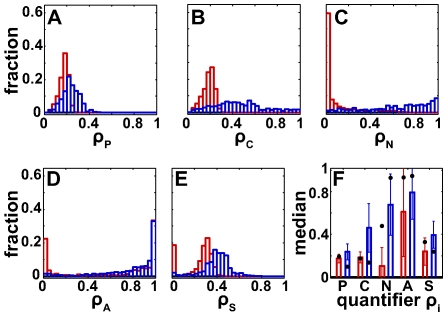
The two-sites model (blue) has greater local robustness than the autocatalytic model (red). Shown are the distributions of (A) robustness to local parameter perturbations 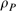, (B) robustness to total concentration perturbations 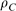, (C) robustness to molecular noise 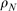, (D) attraction of the cycle 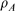, and (E) sensitivity of the period 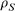. In (F) median values are shown with their associated standard deviation (error bars) for both models and all five quantifiers. Black dots indicate local robustness values for the previously published parameter vector's [31],[32].

Figure 4A shows the distribution of 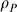, our quantifier of robustness to local parametric perturbations for both the autocatalytic model and the two-sites model. The median robustness of the autocatalytic model is lower by 29% (median 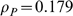 and 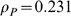 for the autocatalytic and two-sites model, respectively; see table 1 for significance).

Our combination of global and local analysis allows us to ask whether individual chemical reactions (represented through their parameters) are particularly important for a model's robustness. To this end, we investigated whether there exist statistical associations between 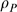 and any of the model parameters. One striking such association stands out for the autocatalytic model (Figure S2A). Specifically, 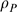 is highly associated with 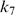, (Spearman's 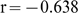), whereas all other parameters and 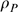 show only 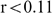 (Spearman's partial correlation given 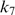). A glance at the model equations (Text S1, section A.4) shows that the reaction associated with 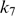 dephosphorylates KaiC* and thus triggers the initialization of a new autocatalytic cycle. If this initialization occurs too fast (at large 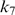), synchronization of complex formation and absorption of perturbations is poor.

As an extension of this quantifier, properly correlated parametric perturbations are used to address the robustness to temperature changes (see *Methods*). We find that the two-sites model has a median robustness only 4% greater than the autocatalytic model (Figure S3B). Individual analyses of both models show why this difference, yet significant (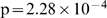), is small compared to the difference in 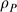. On the first hand, the autocatalytic model is more robust to such correlated perturbations than to uncorrelated perturbations (median of 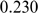, and 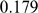, respectively). The large difference between the two cases for the autocatalytic model (Figure S3A and S3B, red bars) can be explained by the strong association between 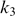 and 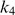 discussed above: correlated perturbations cannot be aligned with the most constrained direction of the viable parameter volume. On the other hand, the two-sites model, which does not have such highly associated parameters, does not show increased robustness to correlated parameter changes (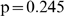).

We next turn to total concentration perturbations 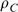 (distribution shown in Figure 4B). Here, the two-sites model is on average 2.5-fold more robust than the autocatalytic model, with a median 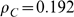 and 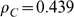 for the autocatalytic and two-sites model, respectively. For instance, for 10% of viable parameter vectors in the two-sites model, more than 80% of perturbations leave the circadian oscillation intact. Exactly as for 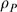, we find that in the autocatalytic model, 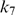 strongly influences 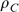 (Figure S2B), with a Spearman's rank correlation between 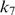 and 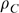 of 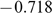, which underscores the importance of this dephosphorylation reaction.

We next assessed robustness 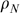 to molecular noise. To this end, we used Gillespie's algorithm [50] to simulate an oscillator with 2000–6000 molecules in a reaction volume of 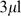, numbers that are of the correct order of magnitude for the number of Kai proteins in a cyanobacterial cell [49]. Here again, the two-sites model is significantly more robust, with a median (mean) value of 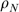 that is 45 (6.5) times larger (Figures 4C and 4F). For example, for the autocatalytic model, fewer than 6% of viable parameter vectors show 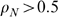 (Figure S2C), whereas more than 80% of the parameters show 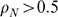 in two-sites model, where noise also affects only a small region of the viable parameter volume (Figure S2D). We discuss in the Text S1, sections B.3 and B.4, that the reactions forming KaiAC, and those forming and destroying 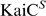 are of particular importance for robustness to molecular noise.

We next turn to the attraction of the cycle 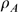, whose distribution is shown in Figure 4D. The two-sites model has a significantly higher median 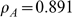 compare to 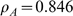 for the autocatalytic model. An analogous difference holds for period sensitivity (Figure 4E), where 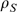 is on average 65 percent greater in the two-sites model.

We had noted previously that 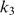 and 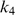 are strongly and negatively associated with global robustness. When analyzing their association with period sensitivity, we find that they also have a strong and opposite impact on the period (results not shown). The reason is the same as discussed in the results for global robustness, namely that the autocatalytic feature that is so central to this model requires a delicate balance of two reactions producing and destroying KaiAC*. This feature also explains the higher robustness to temperature compensation as discussed above.

**Figure 5:**
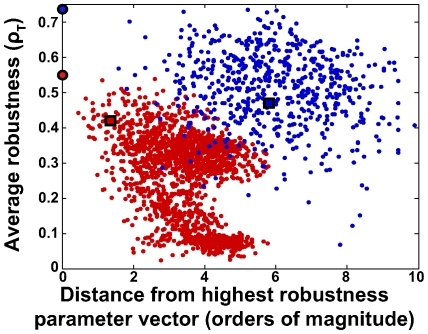
Distribution of the average local robustness 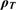 for the two models. For each viable parameter vector 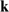, the figure shows its distance (horizontal axis) from the viable parameter vector with the highest average local robustness 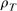 plotted against the 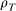 of 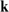 (vertical axis); autocatalytic model (red) and two-sites model (blue). Large circles correspond to the two parameter vectors with the highest 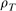 for each model, and squares correspond to published parameter vectors [31],[32]. The greater the distance of 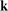 to the most robust parameter vector, the lower its 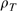. This negative association is stronger for the autocatalytic model.

To summarize, the two-sites model shows significantly greater values in each of the local robustness quantifiers we used (Figure 4F). It is thus not surprising that the average 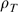 of all five quantifiers also indicates much greater robustness for the two-sites model. For this model, robustness also decreases more slowly with distance from the points of highest average local robustness reflecting a larger volume with high average robustness (Figure 5; see Text S1, section B.5 for details).

## Discussion

Most published work on the robustness of cellular circuits addresses either global or local robustness [17],[18],[20],[29]. Our ‘glocal’ approach overcomes the limitations of both global and local analyses. First, by generating large samples of parameter vectors, the approach can estimate a viable volume of parameter space that yields a behavior of interest. It is thus not easily misled by results derived from a particular chosen point in parameter space, in contrast to parameter fitting that yields only single point estimate. This feature is particularly important for biochemical models that are *structurally* or practically unidentifiable [5],[6],[51],[52]. For the potentially large class of models with this property, model parameters that yield an observed behavior cannot be uniquely identified even in the presence of arbitrarily abundant and error free data. In order to discriminate between possible parameters and models, new experiments could be designed using the results of our robustness analysis. Second, the analysis of parameter vectors spanning multiple orders of magnitude shows how local robustness varies in parameter space. Third, a combination of local and global analyses lends itself to deeper mechanistic insight into circuit behavior. In particular, it can lead to the identification of key parameters important for robustness. Obvious applications include synthetic biology, where tunability of a synthetic circuit's robustness by changing key parameters is highly desirable. Finally, by studying different quantifiers of local robustness, one can obtain trade-offs between robustness and other system properties.

Methods similar to the global part of our approach have been proposed earlier [22],[53]. However, by using principal component analysis, our global method samples more efficiently, a necessity for studying high dimensional parameter spaces.

Potential limitations of our approach include the requirement for a starting parameter vector to initialize global sampling. We used published information for this vector [31],[32]. However, even where such information is unavailable, random sampling and optimization techniques [4] are available to permit creation of such a vector. A second limitation regards the range of the region in parameter space from which one samples. To avoid biased estimation of robustness, the size of this range should be chosen beyond the biophysical bounds on parameters. Note that a conservative choice of this range does not hamper our approach, because our iterative procedure quickly directs the sampling to viable regions.

A third potential limitation regards computational requirements, because our global approach requires numerical integration of a model for hundreds of thousands of parameter vectors, and local robustness estimation for thousands of these vectors. Nonetheless, the approach is feasible with currently available technology. For example, global robustness analysis for the 12-dimensional two-sites model involving nearly 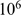 parameter vectors, and 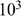 local perturbations for each of the resulting viable vectors executes in less than 5 days on a commercially available eight-core (Intel Xenon X5355 @ 2.66 GHz) architecture. The inevitable exponential scaling of complexity with parameter dimensions can only be mitigated by a guided sampling procedure like ours.

In our application of the method to two circadian oscillator models, we find that the two-sites model shows vastly greater global robustness than the autocatalytic model, with 39-fold and 5-fold allowable parameter variation, respectively, along each parameter dimension on average. Similarly, the two-sites model is also more robust for each of several different quantifiers of local robustness, including robustness to parameter changes, molecular noise, transient state perturbation, and period sensitivity. Based on these considerations alone, the architecture of the two-sites model is superior to the one of the autocatalytic model. If robustness is advantageous, and if this oscillatory mechanism is realizable biochemically [35],[41], it should be the preferred architecture. This observation is consistent with recent experiments that provide strong evidence in favor of ordered phosphorylation in the cyanobacterial clock [47],[54]. In contrast, the autocatalytic mechanism [31], obtained by interpreting experimental results of [49], whereas phosphorylated KaiC facilitates KaiA-KaiC association and subsequent KaiC phosphorylation, was not confirmed by recent experiments [32],[47],[54].

The ‘glocal’ combination of global and local robustness analysis shows which chemical reactions in these models are of particular importance for robustness (or a lack thereof). For example, the rates of two central reactions of the autocatalytic loop in the autocatalytic model need to be delicately balanced, a property that partially accounts for its lack of global robustness. Put differently, the central feature of this model is partly responsible for its low robustness. In the two-sites model, our local analysis shows that the rates of the reactions that form and destroy 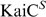 are of particular importance for its robustness. For low values of these parameters, the concentration of 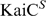 fluctuates to a greater extent. The resulting fluctuations are then amplified by the feedback loop central to this model. In addition to the analysis of these two cyanobacterial circadian models, results obtained with the Goodwin model (see Text S1, section C) show the feasibility of this glocal method for models with a different structure. Our analysis of this generic circadian oscillator also demonstrates the importance of a tight regulation of the feedback component.

In both cyanobacterial models, our evidence suggests that the regions of parameter space where viable parameters occur are connected. This observation is significant to understand how robustness of circadian oscillations could evolve [52],[55],[56], in particular through gradual, small changes of individual parameters. The volume formed by these parameter vectors likely forms a ‘neutral volume’ [57] in which circadian oscillations with a given period and amplitude are preserved. However, what is changing in this volume is local robustness. Thus, if local robustness (or one aspect thereof) is adaptive, then robust circuits are readily accessible to natural selection through the connectedness of the neutral volume, without the need to change the oscillatory behavior itself. In this regard, it is also intriguing to see that the published parameter vectors for either model do not show maximal robustness. If these vectors reflect biological reality, then optimization criteria aside from robustness remain to be discovered, or some unknown constraint may prevent maximization of robustness.

## Methods

The first, global part of our method identifies the viable set 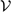 for a given sampled set 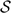 that comprises of the order of 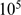 parameter vectors uniformly sampled in some closed region of 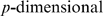 space. We chose the region to be a hyper-cube centered around nominal published values [31],[32], spanning six orders of magnitude for each component. In order to avoid biased estimates the interval bounds should be beyond what is biophysical feasible. Such an a priori range needs to be established, both for practical reasons, and for models that are unidentifiable [5],[51],[52] (see Text S1, section A.1, and Figure S4). The sampling method involves an iterative procedure, which we now describe. In each iterative step 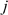 it generates a set 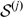, and identifies the viable subset 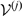. The first set 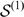 is a Monte Carlo sample of the parameter space obtained via a large (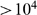) number of 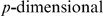 Gaussian random variates, centered on a known viable parameter vector [31],[32]. (Figure 1A). We then determine the viable subset 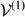 of 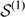, which comprises of the order of 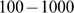 elements in our application. The next step of the procedure consists of a principal component analysis (PCA) of the viable parameter set 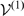. PCA is a technique to identify linear statistical structure in high-dimensional data sets [30]. We use it here to identify associations among viable parameters that can guide our sampling in subsequent iterations. Specifically, the set 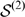 and subsequent sets are generated from previous parameter sets as follows
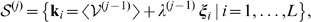
(1)for all 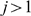, where 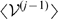 stands for the element-wise mean of parameter vectors in the set 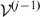 and 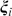 is the 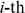 realization of a 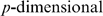 Gaussian process with zero mean and covariance matrix 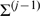. The size of 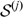 is given by 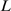 (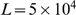 in our application). The entries 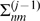 are the pairwise covariances of parameters 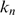 and 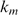 in the set 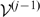. We compute this matrix, whose eigenvectors are the principal axes of the set 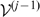, through PCA. The real valued factor 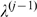 determines the variance of the 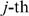 Gaussian process by scaling the standard deviations of the distribution along the PCA directions of the 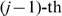 iteration (Figure 1A). In this approach PCA avoids “wasting” sampling effort on parameter regions where viable parameter vectors are not likely to be found. As described thus far, our procedure serves to identify major axes of viable parameter variation for sampling and the dispersion of the viable parameters along them. To establish global measures of robustness, we then perform a Monte Carlo integration (Figure 1B). Specifically, we construct a hyperbox 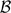 in parameter space whose axes are parallel to the PCA axes of the last iteration. In each dimension, the limits of this box are defined by the most extreme components of the viable parameters found in the last iteration of sampling along these axes. We then generate a set 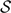 of at least 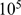 parameter vectors sampled uniformly within 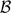, of which some fraction 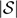 will be viable. An appropriate global measure of robustness for any one model is the *viable volume* 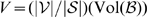, where 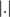 denotes the number of elements in a set. The rationale behind this measure is that with increasing robustness 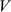, a perturbation of a parameter or parameter vector is increasingly likely to generate another viable parameter vector. To compare models with different number of parameters, we define the normalized viable volume as robustness 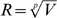. Note that it would not be appropriate to just consider the ratio 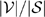 as a robustness measure when comparing models (Figure S5). The main functions for this analysis, written in MATLAB, are available for download at http://www.bioc.uzh.ch/wagner/publications-software.html.

To estimate the sampling errors in the viable fractions and volumes, we note that 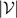, as estimated by Monte Carlo integration is a binomially distributed random variable [30],[58]. An estimate of its standard deviation is 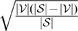. Of interest is the coefficient of variation or relative error, defined as the standard deviation divided by the mean. For 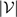, this relative error is given by 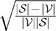. For the normalized quantity 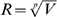, the relative error needs to be divided by 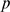, i.e., it calculates as
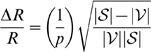
which scales as 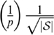. Furthermore we estimate the necessary sample size 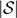 for a given relative accuracy 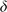 and confidence. Applying Hoeffding's inequality [59], and exploiting the fact the random variables are binomally distributed, we obtain
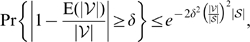
where 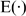 denotes the expectation operator. Thus, estimating the sampling *acceptance ratio* 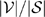 from a sufficiently large ensemble and assuming it to be constant for the successive sampling, we can compute a lower bound for the necessary sample size. For example, asking for 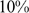 accuracy with a confidence of 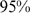 at an acceptance ratio of 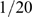, Hoeffding's bound requires the sample size to be 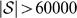.

We now briefly comment on how estimation errors scale with the number of dimensions 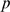. The only possible general statement is that the ratio between the viable volume and the volume of the sampling box scale exponentially with 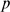. Therefore, 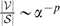 with 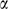 being dependent on the geometry of the viable volume. For example, 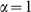 if the viable volume is identical to the sampling hyper-rectangle, and only in this trivial case does the error not depend on 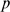. If the viable parameter volume has an ellipsoidal shape, and if the dimension increases from 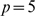 to 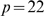, then 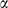 increases from 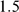 to 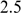. The coefficient of variation (relative error) of the viable volume scales as 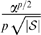. The size and the shape of the sampling hyper-rectangle is crucial for low errors: a larger hyperbox means that an exponentially greater number of points needs to be sampled for high dimensional systems to ensure constant error. These observations underscore the usefulness of PCA, which can dramatically reduce computational requirements.

The second, local part of our method assesses the robustness of every viable parameter 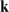 in terms of five quantifiers. The first local robustness quantifier 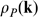 computes to the fraction of local random perturbations of parameters that preserve 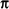. A perturbation is generated by multiplying all parameter values with uncorrelated Gaussian variates of variance 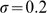 and mean 1. To address the robustness to temperature changes the Arrhenius equation has to be used ideally [8],[31],[46]. However, this approach requires knowledge of the activation energies of each reaction in a system, which is usually not available. We thus simply assume that an increase in temperature corresponds to an random increase of all parameters. This aspect of robustness is quantified with the same approach used for estimating 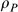. Mean and standard deviation are the same, but perturbations are correlated, such that all parameters are multiplied with variates that are either above one or below one for a particular perturbation. The second local robustness quantifier 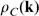 regards alterations in the total amount of key proteins. For example, the *in vitro* reconstitution of the cyanobacterial circadian oscillator uses a pre-determined number of the Kai molecules [37],[60]. This number may vary *in vivo*, for example due to changes in cell volume caused by the cell division cycle. To estimate 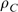, we generate a large number of perturbed concentrations, and numerically integrate the model with these perturbed concentrations. For a given parameter vector 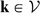, we define 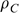 as the fraction of these perturbations preserving 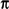. The third robustness quantifier, 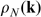, reflects that chemical reactions are stochastic events [12],[20],[61]. To quantify robustness to such molecular noise, we perform many stochastic simulations [50], and define for each viable 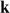, 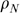 as the fraction of trajectories that preserve 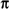. The fourth robustness quantifier, 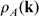 (for attraction of the cycle), measures how fast the oscillator returns to its cycling behavior when its trajectory is transiently perturbed with the use of Floquet multipliers. The fifth quantifier, 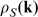 (for sensitivity analysis of period), assesses the effect of an infinitesimal change of an individual parameter or parameter vector on the period of a model. The larger the value of 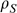, the more robust a model is. A value of 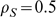 means that a one percent change in a parameter vector results in a one percent change in the period. The last two quantifiers are specific to systems involving stable oscillations. For the full mathematical details on these five quantifiers see Text S1, section A.3.

## Supporting Information

Figure S1 Viable parameter sets form large connected regions in parameter space. (A) Autocatalytic model, (B) two-sites model. Pairs of viable parameter vectors (black dots) are connected by blue lines, if they are likely to be part of the same connected region of parameter space, as determined by numerical analysis explained in the text. Parameter vectors that cannot be connected to other parameter vectors are shown as red dots. The graph is shown as a projection on to the axes formed by k_5_ and k_6_ for (A), and as a projection onto the axes formed by k_1_ and k_2_ in (B), because these projections best illustrate that the viable region is not convex.
(0.52 MB PDF)

Figure S2 Correlations of the local robustness quantifiers with model parameter. (A) Parameter k_7_ (horizontal axis) negatively affects robustness to parameter perturbations (vertical axis) in the autocatalytic model (Spearman's r = −0.638, p<10^−323^, n = 1828). (B) Parameter k_7_ (horizontal axis) negatively affects robustness to parameter perturbations (vertical axis) in the autocatalytic model (Spearman's r = −0.718, p = 2.81×10^−289^, n = 1828). (C) Score for robustness to molecular noise for the autocatalytic model plotted against k_1_ and (D) the two-sites models plotted against k_2_. In the autocatalytic model, k_1_ has a Spearman's correlation coefficient with ρ_N_ of 0.921 (p<10^−323^, n = 1828) and less that 6 percent of the parameter vectors have a score above 0.5. For the two-sites model, k_2_ has a correlation coefficient with ρ_N_ of 0.629 (p<10^−323^, n = 604) and more than 80 percent of the parameter vectors have a score above 0.5.
(0.87 MB PDF)

Figure S3 (A) Distribution of the scores for the robustness to parameter perturbations (autocatalytic model in red and two-sites model in blue), similar as Figure 4B. (B) Distribution of the scores for the robustness to temperature changes. The results are obtained with the same algorithm as the one for ρ_P_ but the random variates are correlated such that for a particular perturbation all parameters are either increased or decreased. In this case, the median robustness for the two-sites model is only 4 percent larger than the median of the autocatalytic model (p = 2.28×10^−4^, Wilcoxon rank sum test).
(0.09 MB PDF)

Figure S4 Illustration of the proposed sampling approach based on interval constraints. The a priori sampling range (light-gray) and two systemic properties π_1_ and π_2_ allowed to assume values in predetermined intervals induce constraints in parameter space and partition it into regions that are viable and those that are not. The parameter region preserving π_2_ is unbounded, accounting for the situation of unidentifiability and indicates the necessity for an a priori sampling range.
(0.27 MB PDF)

Figure S5 The importance of incorporating volume information in estimating global robustness. One might argue that it would be sufficient to just use the ratio C = |*V*|/|*S*| as a measure of global robustness. This is not the case if one wants to compare models where both the geometry and the size of a model's viable set vary among models. The reason is that the geometry of the viable volume critically influences C. The Figure shows the shape of viable sets and the circumscribed hyperbox for three hypothetical models. The viable sets in (A) and (C) have very different shapes, but fit into a hyperbox of the same size. If these models are compared, the size of the hyperbox would therefore be irrelevant (and one would say that the model of (A) has greater robustness than the model of (B)). The viable sets in (A) and (B) have the same geometry but the viable set of (B) can be circumscribed by a smaller hyperbox. The ratio C would be the same for these two models. The models in (B) and (C) have both a different geometry and extension. In that case the differing box volumes must be taken into account, and the expression V = (|*V*|/|*S*|)⋅Vol(*B*) accomplishes that. Put differently, it would be appropriate to use the ratio C only if parameter sets were to be sampled from boxes of the same size for different models, an approach that we avoid, because it would lead to very large errors in the Monte Carlo integration for some models.
(0.31 MB PDF)

Text S1 Supplementary methods, supplementary results, robustness analysis of the Goodwin model and supplementary figures S6 to S11.
(2.41 MB PDF)
