# Supplementary figures and images for: ‘Glocal’ Robustness Analysis and Model Discrimination for Circadian Oscillators
Source: PLoS Comput Biol. 2009 Oct 16;5(10):e1000534. doi: 10.1371/journal.pcbi.1000534 (PMC2758577; doi:10.1371/journal.pcbi.1000534)

**A**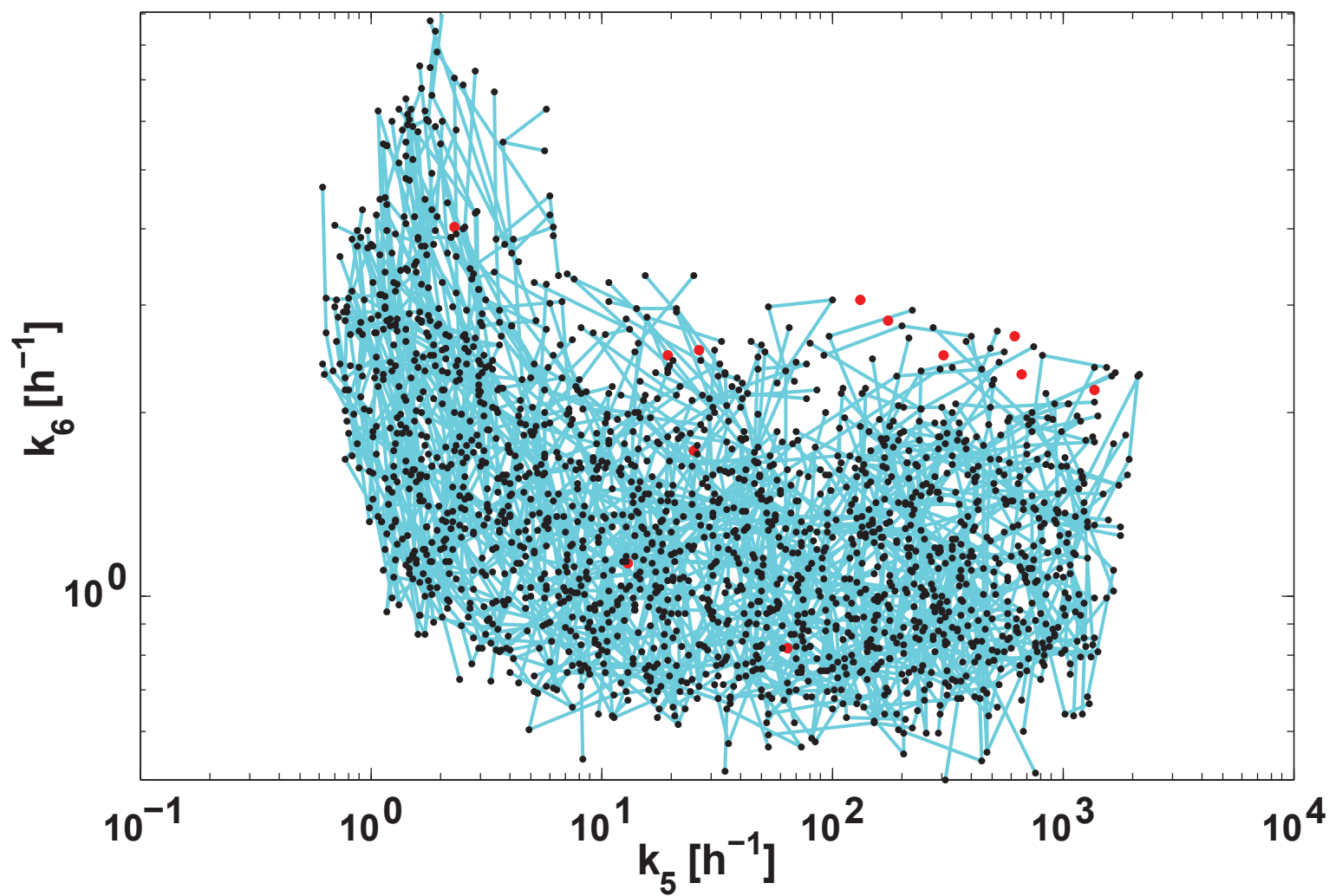**B**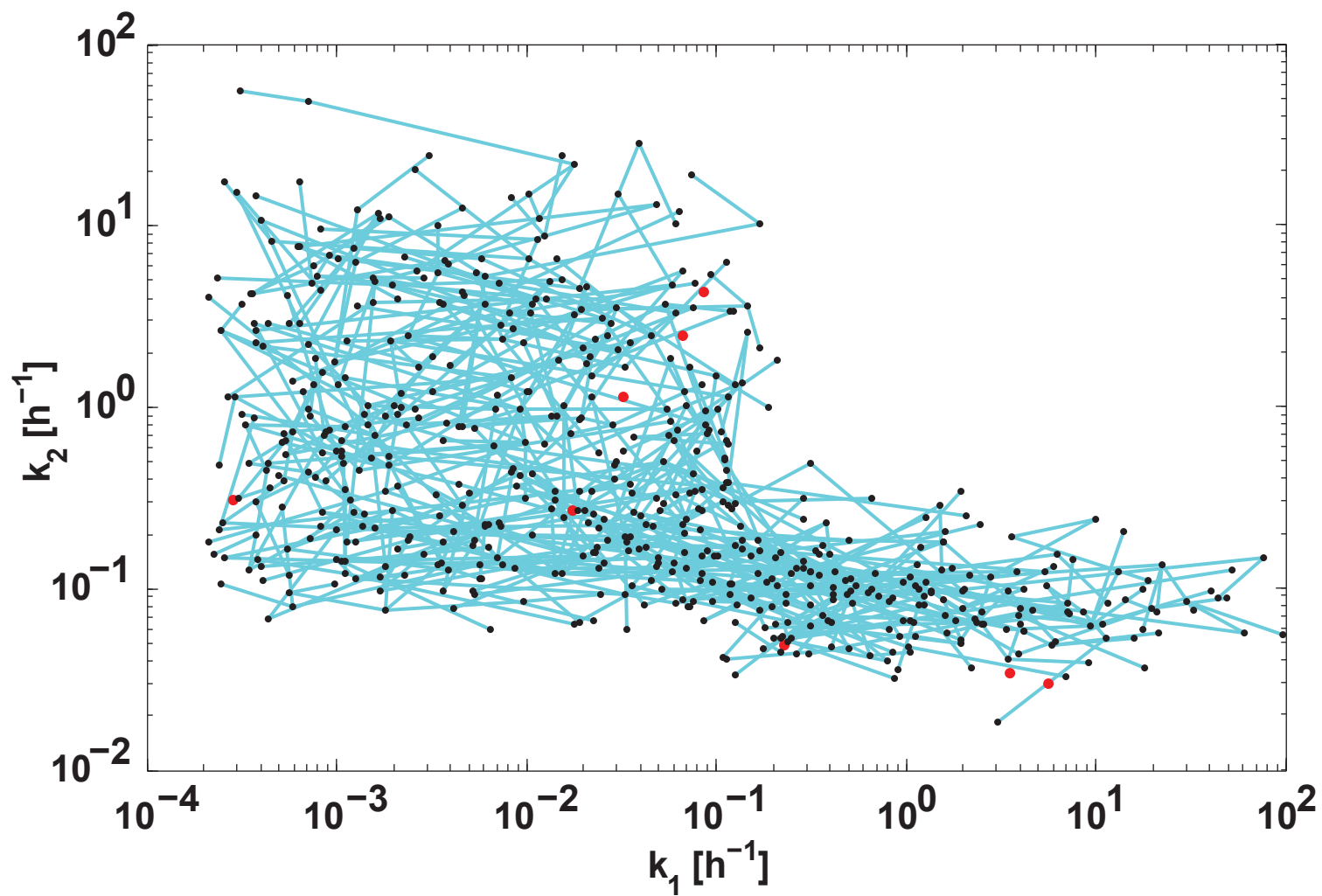

Supplement: Figure S1 — Viable parameter sets form large connected regions in parameter space. (A) Autocatalytic model, (B) two-sites model. Pairs of viable parameter vectors (black dots) are connected by blue lines, if they are likely to be part of the same connected region of parameter space, as determined by numerical analysis explained in the text. Parameter vectors that cannot be connected to other parameter vectors are shown as red dots. The graph is shown as a projection on to the axes formed by k5 and k6 for (A), and as a projection onto the axes formed by k1 and k2 in (B), because these projections best illustrate that the viable region is not convex. (0.52 MB PDF) [file pcbi.1000534.s001.pdf]

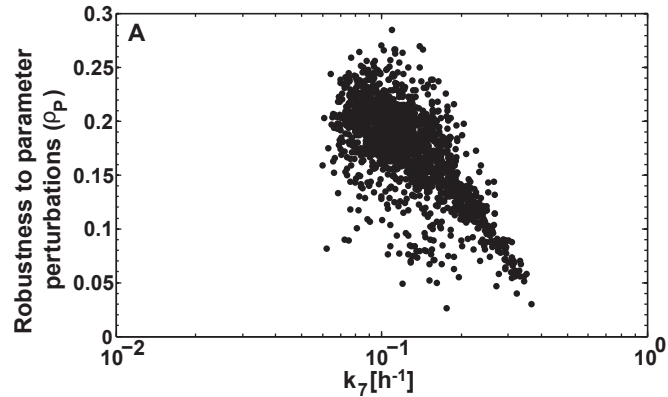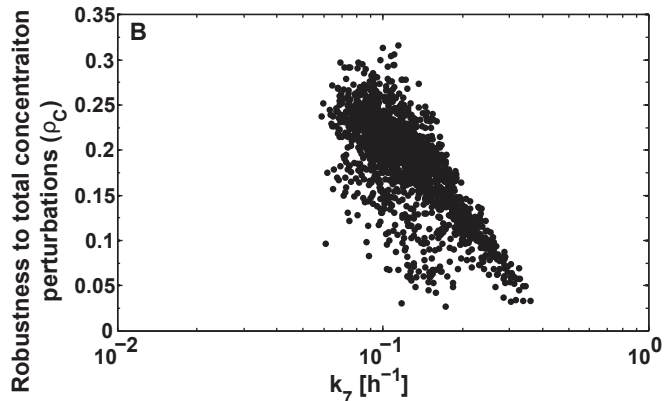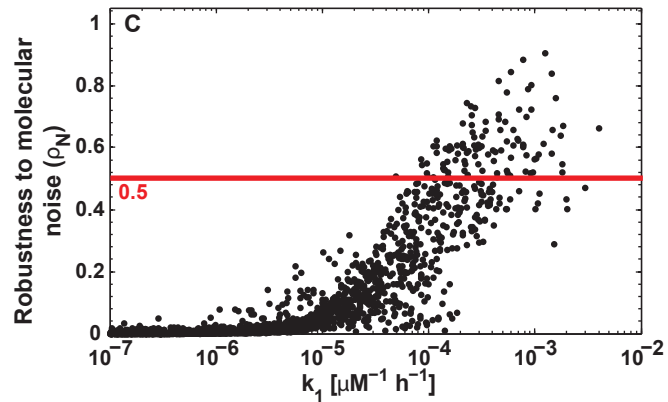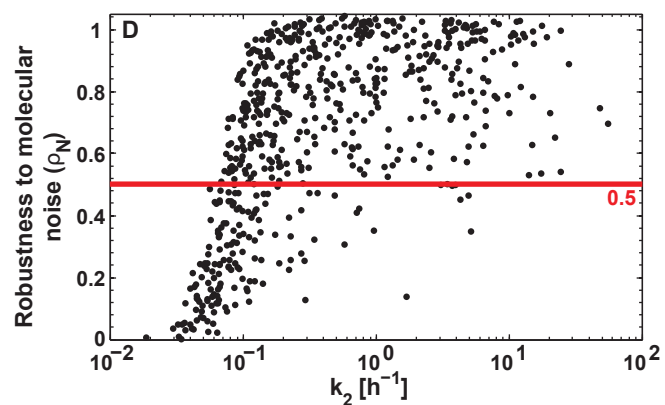

Supplement: Figure S2 — Correlations of the local robustness quantifiers with model parameter. (A) Parameter k7 (horizontal axis) negatively affects robustness to parameter perturbations (vertical axis) in the autocatalytic model (Spearman's r = −0.638, p<10−323, n = 1828). (B) Parameter k7 (horizontal axis) negatively affects robustness to parameter perturbations (vertical axis) in the autocatalytic model (Spearman's r = −0.718, p = 2.81×10−289, n = 1828). (C) Score for robustness to molecular noise for the autocatalytic model plotted against k1 and (D) the two-sites models plotted against k2. In the autocatalytic model, k1 has a Spearman's correlation coefficient with ρN of 0.921 (p<10−323, n = 1828) and less that 6 percent of the parameter vectors have a score above 0.5. For the two-sites model, k2 has a correlation coefficient with ρN of 0.629 (p<10−323, n = 604) and more than 80 percent of the parameter vectors have a score above 0.5. (0.87 MB PDF) [file pcbi.1000534.s002.pdf]

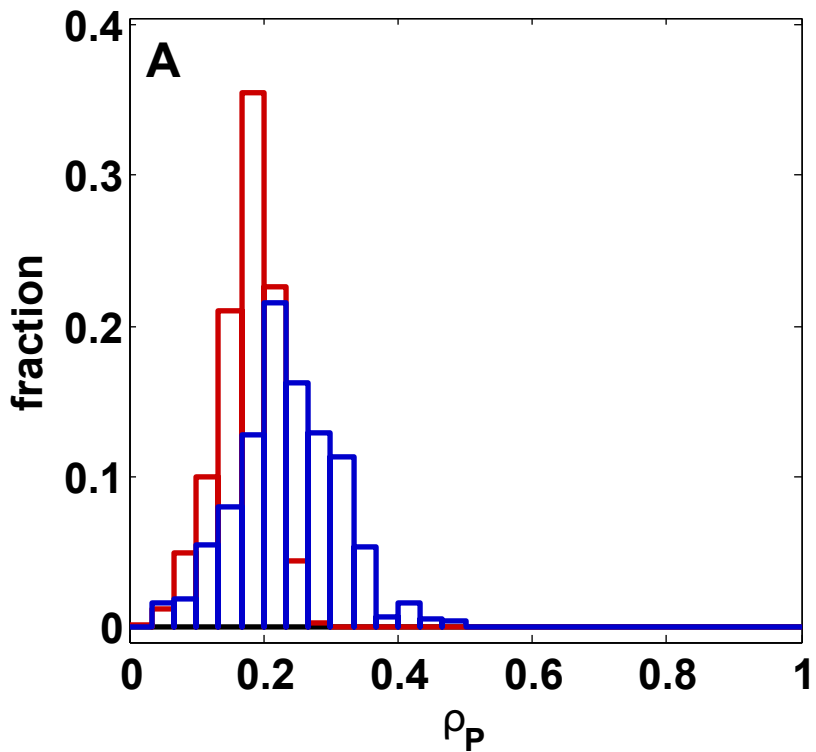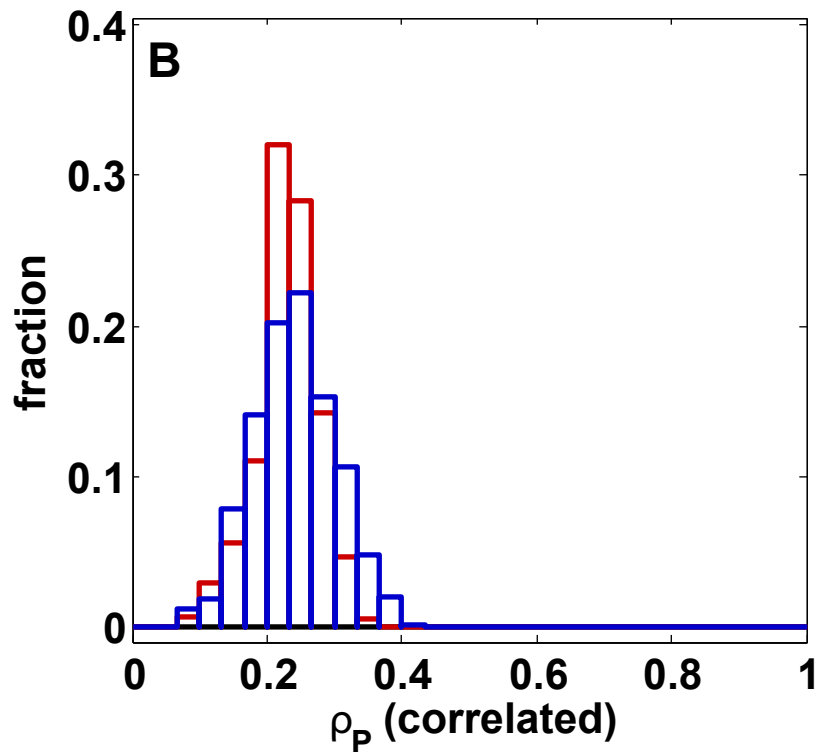

Supplement: Figure S3 — (A) Distribution of the scores for the robustness to parameter perturbations (autocatalytic model in red and two-sites model in blue), similar as Figure 4B. (B) Distribution of the scores for the robustness to temperature changes. The results are obtained with the same algorithm as the one for ρP but the random variates are correlated such that for a particular perturbation all parameters are either increased or decreased. In this case, the median robustness for the two-sites model is only 4 percent larger than the median of the autocatalytic model (p = 2.28×10^−4^, Wilcoxon rank sum test). (0.09 MB PDF) [file pcbi.1000534.s003.pdf]
