# Supplementary material for: ‘Glocal’ Robustness Analysis and Model Discrimination for Circadian Oscillators
Source: PLoS Comput Biol. 2009 Oct 16;5(10):e1000534. doi: 10.1371/journal.pcbi.1000534 (PMC2758577; doi:10.1371/journal.pcbi.1000534)
Supplement: Figure S4 — Illustration of the proposed sampling approach based on interval constraints. The a priori sampling range (light-gray) and two systemic properties π1 and π2 allowed to assume values in predetermined intervals induce constraints in parameter space and partition it into regions that are viable and those that are not. The parameter region preserving π2 is unbounded, accounting for the situation of unidentifiability and indicates the necessity for an a priori sampling range. (0.27 MB PDF) [file pcbi.1000534.s004.pdf]

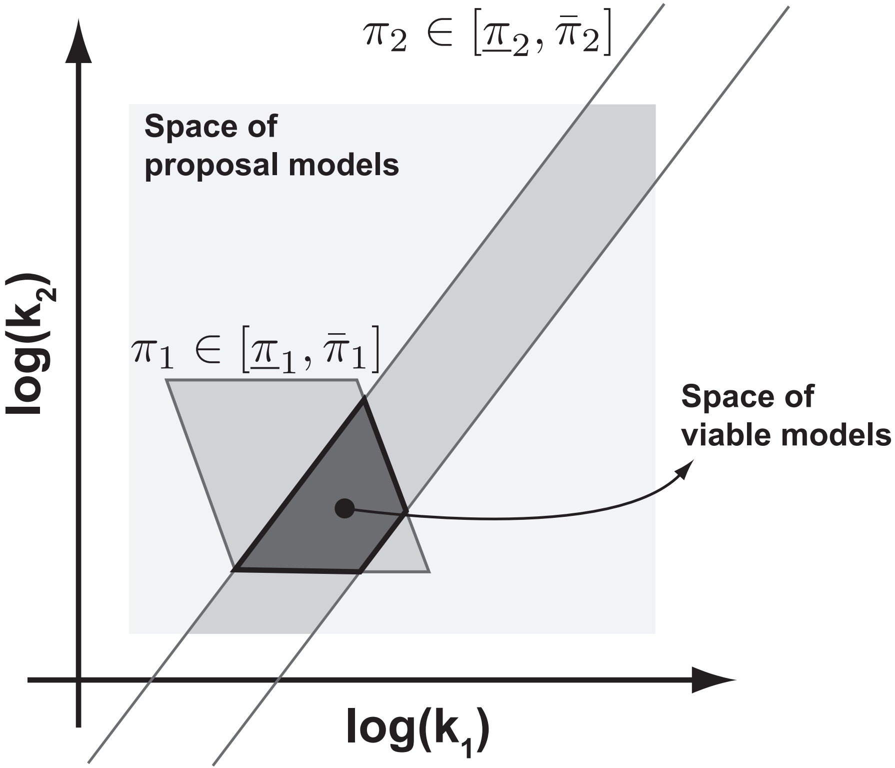
$\pi_2 \in [\underline{\pi}_2, \bar{\pi}_2]$
Space of proposal models
$\pi_1 \in [\underline{\pi}_1, \bar{\pi}_1]$
Space of viable models
log(k$_2$)
log(k$_1$)
