# Supplementary material for: ‘Glocal’ Robustness Analysis and Model Discrimination for Circadian Oscillators
Source: PLoS Comput Biol. 2009 Oct 16;5(10):e1000534. doi: 10.1371/journal.pcbi.1000534 (PMC2758577; doi:10.1371/journal.pcbi.1000534)
Supplement: Figure S5 — The importance of incorporating volume information in estimating global robustness. One might argue that it would be sufficient to just use the ratio C = |V|/|S| as a measure of global robustness. This is not the case if one wants to compare models where both the geometry and the size of a model's viable set vary among models. The reason is that the geometry of the viable volume critically influences C. The Figure shows the shape of viable sets and the circumscribed hyperbox for three hypothetical models. The viable sets in (A) and (C) have very different shapes, but fit into a hyperbox of the same size. If these models are compared, the size of the hyperbox would therefore be irrelevant (and one would say that the model of (A) has greater robustness than the model of (B)). The viable sets in (A) and (B) have the same geometry but the viable set of (B) can be circumscribed by a smaller hyperbox. The ratio C would be the same for these two models. The models in (B) and (C) have both a different geometry and extension. In that case the differing box volumes must be taken into account, and the expression V = (|V|/|S|)⋅Vol(B) accomplishes that. Put differently, it would be appropriate to use the ratio C only if parameter sets were to be sampled from boxes of the same size for different models, an approach that we avoid, because it would lead to very large errors in the Monte Carlo integration for some models. (0.31 MB PDF) [file pcbi.1000534.s005.pdf]

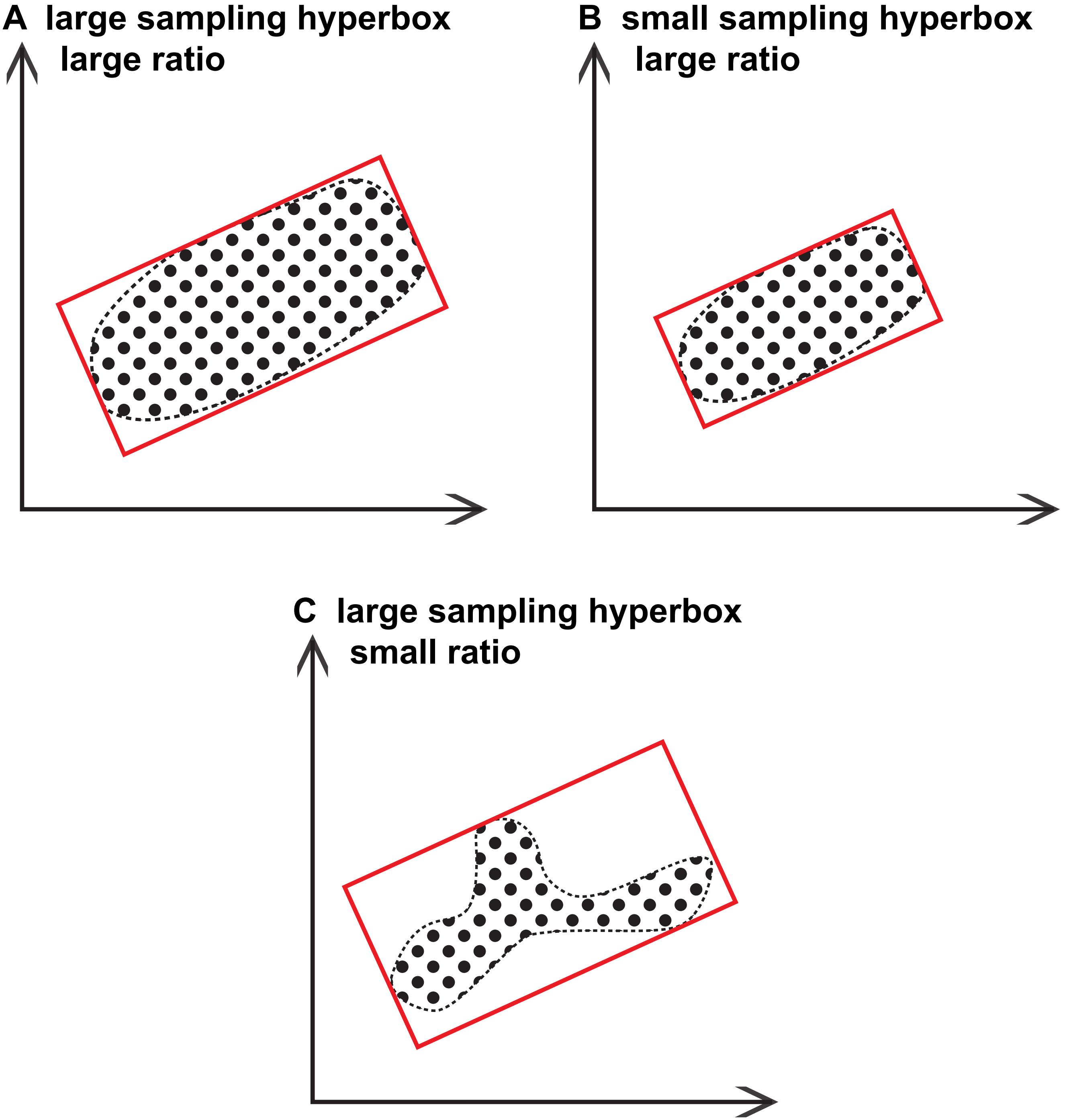
A large sampling hyperbox
large ratio
B small sampling hyperbox
large ratio
C large sampling hyperbox
small ratio
