# Supplementary material for: ‘Glocal’ Robustness Analysis and Model Discrimination for Circadian Oscillators
Source: PLoS Comput Biol. 2009 Oct 16;5(10):e1000534. doi: 10.1371/journal.pcbi.1000534 (PMC2758577; doi:10.1371/journal.pcbi.1000534)
Supplement: Text S1 — Supplementary methods, supplementary results, robustness analysis of the Goodwin model and supplementary figures S6 to S11. (2.41 MB PDF) [file pcbi.1000534.s006.pdf]

Supplementary Information of the article 'Glocal' Robustness Analysis and Model Discrimination for Circadian Oscillators

Marc Hafner[1,2,3], Heinz Koeppl[1,4], Martin Hasler[1] Andreas Wagner[2,3,5,6,*]
1 School of Computer and Communication Sciences, Ecole Polytechnique Fédérale de Lausanne (EPFL), CH-1015 Lausanne, Switzerland
2 Department of Biochemistry, University of Zurich, CH-8057 Zurich, Switzerland
3 Swiss Institute of Bioinformatics, CH-1015 Lausanne, Switzerland
4 Plectix Biosystems, Somerville, MA 02144, USA
5 The Santa Fe Institute, Santa Fe, NM 87501, USA
6 Department of Biology, University of New Mexico, Albuquerque, NM 87131, USA
∗ E-mail: aw@bioc.unizh.ch

A Methods

A.1 Introduction

The proposed sampling approach is complementary to traditional approaches such as model calibration and has the following conceptual underpinning. The structure and parameters of most reported models are underdetermined with respect to the available experimental observations [1]. Moreover, some biochemical models are *structurally* unidentifiable [2]. That is, even in the presence of arbitrarily abundant and error free data, model parameters that yield the observed behavior cannot be uniquely identified. However, model calibration is designed to find the unique parameter vector that renders the model behavior the closest to the experimentally observed behavior. Consequently, often the single point-estimate for a model parameter, returned by a calibration procedure, may contain little information about the underlying biophysical process. The sampling approach alleviates this implicit degeneracy by making it explicit and instead returns *all* parameter vectors that are consistent with the observed behavior.

Model behavior may be characterized by a collection of *systemic properties* $\boldsymbol{\pi}(\mathbf{k})$ assuming values within a predetermined range, i.e., $\boldsymbol{\pi}(\mathbf{k}) \in [\underline{\boldsymbol{\pi}}, \overline{\boldsymbol{\pi}}]$, a hyper-rectangle. We then say the properties $\boldsymbol{\pi}$ are *preserved* under parameter vector $\mathbf{k}$. In our application for instance, these properties are oscillation amplitude and frequency of phosphorylated KaiC, that are required to stay within a 10% interval around their nominal values [3, 4]. Any such constraint on a systemic property partitions the parameter space into regions that are *viable* and those that are not. Augmenting the collection $\boldsymbol{\pi}$ with a new property and its constraint generally causes the viable region to shrink.

Properties can take various forms. In calibrating a model to experimental time course data for instance, one may want to determine parameter regions exhibiting trajectories that stay within a predefined interval around the experimental time course reflecting the uncertainty of data acquisition. Constraints like those just mentioned are interval constraints rather than optimality criteria as found in optimization techniques such as model calibration (see Figure S4 for an illustration of this approach). This *semi-quantitative* approach is particularly suitable to deal with the ubiquitous uncertainty of biological information, and it can leverage principles from interval analysis [5], semi-quantitative reasoning [6] and robust control theory [7]. Besides its application to robustness analysis the approach may be used to more rigorously account for issues related to identifiability and measurement uncertainty in model calibration.

A.2 Details about global robustness analysis

In our application of the iteration procedure, random variates in the first iteration are uncorrelated, and have a standard deviation of 0.25 in the logarithmic domain, which ensures that this initial sampling covers at least one order of magnitude for each parameter. In subsequent iterations, we found it useful to decrease the variance scaling parameter λ from equation 1 (main text) linearly from $\lambda^{(1)} = 2.5$ to $\lambda^{(5)} = 1.5$, and to keep $\lambda^{(j)}$ constant thereafter. The iterations can be carried out until convergence (e.g. until the PCA axes in subsequent deviate from each other by less than a pre-specified angle), or for a fixed number of steps, in order to amass a sufficient number of viable parameter vectors in $\mathcal{V}$.

Different models may have different dimensions p. For example, the two circadian oscillator models we study differ in this respect. (The autocatalytic model has 8 state variables and 7 parameters, and the two-sites model has 4 state variables and 12 parameters.) To allow model comparison, we need to normalize our measure of global robustness to account for these differences. If along each (principal component) axis of parameter space the same fraction c of randomly chosen parameters were to preserve the properties $\boldsymbol{\pi}$, then the ratio $C = |\mathcal{V}|/|\mathcal{S}| = c^p$, and $c = \sqrt[p]{C}$. Although c is almost certain to vary among different axes, $\sqrt[p]{C}$ can still be thought of as a per-parameter robustness of a model. This observation motivates the normalized robustness measure $R = \sqrt[p]{V} = \sqrt[p]{(|\mathcal{V}|/|\mathcal{S}|)\mathrm{Vol}(\mathcal{B})}$. In the log-domain, a value of $R = 0.5$ ($V = 0.5^p$) means that on average we can change every parameter over half an order of magnitude (by around 32%) while preserving $\boldsymbol{\pi} \in [\underline{\boldsymbol{\pi}}, \overline{\boldsymbol{\pi}}]$.

A.3 Local robustness quantifiers

A.3.1 Robustness ρ_P and ρ_C

To estimate $\rho_P(\mathbf{k})$ for any specific model, one generates many random perturbations of each viable parameter vector $\mathbf{k}$, for example with a Gaussian distribution centered on $\mathbf{k}$, determine the model's behavior with these parameters, and define ρ_P as the fraction of perturbations preserving $\boldsymbol{\pi} \in [\underline{\boldsymbol{\pi}}, \overline{\boldsymbol{\pi}}]$. The standard deviation of the Gaussian distribution is best chosen such that (i) all values of ρ_P in the allowed interval $[0, 1]$ are observed, and that (ii) ρ_P can be distinguished most significantly for the two considered models.

For our application to the autocatalytic and two-sites model, we perturbed each viable parameter vector $\mathbf{k}$ 1000 times. In each of these perturbations, we multiplied each component k_i of $\mathbf{k}$ with a Gaussian random variate of mean one and standard deviation $\sigma = 0.2$. We chose this value, because it yields different values of ρ_P for different parameter vectors, thus allowing us to assess how robustness varies in different regions of parameter space, and because it permits discrimination of ρ_P among the two models (Figure S8). We took the same approach for the robustness to total concentration perturbations ρ_C.

A.3.2 Robustness to molecular noise, ρ_N

When stochasticity is involved, the definition of a period becomes problematic, because considerable amplitude variations from one period to the next may be present. The Hilbert transform method, which yields information about the phase of a trajectory [8,9] helps us circumvent this problem. It is preferable to the Fourier transform in situations where period needs to be estimated independently from amplitude. To quantify $\rho_N(\mathbf{k})$ we first simulate [10] stochastic trajectories over a large number of periods using $\mathbf{k}$ with a specific cell volume v (see below). We define ρ_N as the ratio of the number of completed cycles with the correct period to the total number of completed cycles in a deterministic simulation of the same

duration.

To quantify robustness to molecular noise, we perform stochastic simulations [10] over some time span τ with a number of molecules determined by a cellular volume $v = 3\mu l$ and published molecular concentrations [3, 4]. For the autocatalytic model (SI A.4), the total numbers of molecules become 5420 for KaiA, 1807 for KaiB, and 6323 for KaiC. For the two-sites model (SI A.5), we obtain analogously 2349 molecules of KaiA and 6142 molecules of KaiC. These values are within ranges observed for cyanobacteria [11]. When changing the cellular volume, the results stay qualitatively the same (results not shown).

To define our robustness measure ρ_N, we denote the desired or empirically observed period of an oscillatory system as T_0. This period is allowed to vary over a certain range (10% for our application). Also, we denote as $\mathcal{T}(\mathbf{k})$ the set of observed period durations in the stochastic simulation of duration τ, for a circuit with parameter vector $\mathbf{k}$. With this notation, we define $\rho_N = t(\mathbf{k})/z(\mathbf{k})$, where $t(\mathbf{k})$ is the total number of cycles with a period in the allowable range that occurred in the time τ, and z is a normalization constant defined as

$$z(\mathbf{k}) = \mathbf{max}\left\{\frac{\tau}{T_0}, |\mathcal{T}(\mathbf{k})|\right\}.$$

The constant $z(\mathbf{k})$ is chosen such that ρ_N is smaller than one and that we avoid misclassification of trajectories having long duration without oscillation, and bursts of oscillations with the correct period. In the following, we will motivate our choice of this normalization by simple examples. Consider a hypothetical oscillator with a desired period of $T_0 = 22h$, 10% allowable variation around this period, and a simulation length $\tau = 330h$. In a deterministic (noiseless) simulation, the oscillator would complete exactly $\frac{\tau}{T_0} = 15$ cycles, but noise could change this number. Typically, it would cause the periods of the cycles to spread over a range larger than allowable, but the majority of cycles might have the correct period. If, for example, we observe 14 cycles, six of them with a period of $22.5h$, six with a period of $23.5h$ and two with a period of $26h$ (outside the allowable range), then ρ_N would calculate as $\rho_N = \frac{12}{15} = 0.8$. The second example concerns the special case where noise speeds up the oscillation. If we observe 16 cycles of period $20.5h$, then the maximum in the normalization function above is important to have ρ_N contained between 0 and 1, because $\rho_N = \frac{16}{max(15,16)} = 1$. Lastly, we consider the case where molecular noise generates pauses in the cycle. We can imagine a trajectory starting with two cycles having an acceptable duration of $22h$, then a long cycle (pause) of $240h$ and again 2 cycles with acceptable period. In this case ρ_N calculates as $\frac{4}{15} = 0.267$ with our normalization. The much simpler normalization that divides by the number of completed cycles would yield an artificially inflated value of $\rho_N = \frac{4}{5} = 0.8$.

In our simulations, we estimate ρ_N as averages over 80 independent simulations over $\tau = 100 \cdot T_0$ hours where T_0 is the duration of the period of each model with its nominal published values [3, 4], i.e., approximately 2200 hours for each model.

A.3.3 Attraction of the cycle, ρ_A

In biological systems, most regular oscillations are limit cycle oscillations [12], where a system returns to its pre-perturbation nominal oscillatory behavior after a perturbation of its trajectory. Some such oscillations may be much more robust, in the sense that they would converge very rapidly to this nominal behavior, whereas others may take a long time to ‘absorb’ the effects of a transient perturbation.

A commonly used analytical approach of estimating this convergence to oscillatory behavior uses Floquet theory [13]. Briefly, a system’s convergence to a cycle can be estimated through its largest Floquet multiplier, which can be thought of the fraction of an small trajectory perturbation that remains after one cycle. For the systems we study, Floquet multipliers assume values between zero and one, and the smaller the multiplier, the faster a perturbation is absorbed. To calculate the largest Floquet

multiplier in our application, we integrate over one cycle the variational equations [13] of the models that we study, and find its largest eigenvalue μ. We define $\rho_A(\mathbf{k}) = 1 - \mu$. This measure of robustness corresponds to the fraction of the perturbation that is 'absorbed' by the system after one cycle. This quantifier is complementary to ρ_N for the following reasons. First, ρ_A is a deterministic measure and thus remains useful for very large molecule copy numbers. Second, it characterizes a system's response to arbitrary transient state perturbations, not just to fluctuations due to molecular noise.

A.3.4 Sensitivity of the period, ρ_S

A measure for the sensitivity of oscillatory behavior assesses the effect of an infinitesimal change of an individual parameter or parameter vector on the period T of a system. Specifically, we estimate the gradient-vector $\partial T/\partial \mathbf{k}$. A component i of this vector with large absolute value indicates a parameter k_i that affects T to a great extent. We define

$$\rho_S(\mathbf{k}) := \left(1 + \left\| \frac{\partial \log(T)}{\partial \log(|\mathbf{k}|)} \right\| \right)^{-1}$$

as our robustness measure.

The logarithm in this expression occurs, because we are interested in the relative effect of one parameter on the period. The expression $\|\mathbf{x}\|$ denotes the Euclidian norm $\sqrt{\sum_i x_i^2}$ of the vector $\mathbf{x}$. The quantifier ρ_S assumes values between zero and one.

In order to estimate ρ_S, we need to derive the first order approximation of the quantity $\frac{\partial T}{\partial k_i}$, i.e., the response of a dynamical systems period T to an infinitesimal change in one component k_i of its parameter vector $\mathbf{k}$. For simplicity of notation, we first derive the expression for a single parameter k and then extend it to a parameter vector $\mathbf{k}$.

For an ordinary differential system $\frac{d\mathbf{x}}{dt} = \mathbf{F}(\mathbf{x}(t), k)$ with a parameter k that has a periodic solution $\boldsymbol{\xi}(t,k) = \boldsymbol{\xi}(t + T(k), k)$ of period $T(k)$, we define the nominal parameter k_0, the corresponding nominal solution $\boldsymbol{\xi}_0(t) = \boldsymbol{\xi}(t, k_0)$ and the nominal period $T_0 = T(k_0)$. The infinitesimal increment of the parameter is dk. With this notation, we can write $dT = T(k_0 + dk) - T_0$ and $d\boldsymbol{\xi}(t) = \boldsymbol{\xi}(t, \mathrm{k}_0 + dk) - \boldsymbol{\xi}_0(t)$. We have a family of solutions $\boldsymbol{\phi}(t, \mathbf{x}_0, k) = \mathbf{x}(t, k)$, with $\mathbf{x}(0) = \mathbf{x}_0$. Then the sensitivity matrix $\mathbf{M}(t)$ can be defined as $(\mathbf{M}(t))_{ij} = \frac{\partial \phi_i}{\partial x_j}(t, \mathbf{x}_0, k)$ and the parameter sensitivity matrix is $(\mathbf{V}(t))_i = \frac{\partial \phi_i}{\partial k}(t, \mathbf{x}_0, k)$.

$\mathbf{M}(t)$ is the solution of the variational equation

$$\frac{d\mathbf{M}}{dt} = \frac{d\mathbf{F}}{d\mathbf{x}}(\xi_0(t), k_0)\mathbf{M}(t) \ , \quad \mathbf{M}(0) = \mathbf{I}$$

and $\mathbf{V}(t)$ the solution of the variational equation

$$\frac{d\mathbf{V}}{dt} = \frac{d\mathbf{F}}{d\mathbf{x}}(\xi_0(t), k_0)\mathbf{V}(t) + \frac{\partial \mathbf{F}}{\partial k}(\xi_0(t), k_0), \quad \mathbf{V}(0) = \mathbf{0}$$

Then up to the first order approximation

$$d\boldsymbol{\xi}(t) \cong \mathbf{M}(t) d\boldsymbol{\xi}(0) + \mathbf{V}(t) dk$$

For simplicity, choose H the Poincaré section as the hyperplane that goes through $\boldsymbol{\xi}_0(0)$ and is perpendicular to $\mathbf{e} = \frac{d\boldsymbol{\xi}_0}{dt}(0) \Big/ \left\| \frac{d\boldsymbol{\xi}_0}{dt}(0) \right\|$. Then $\mathbf{P} = \mathbf{I} - \mathbf{e}\ \mathbf{e}^T$ is the orthogonal projection on the hyperplane H.

Note that by definition $\boldsymbol{\xi}_0(0) = \boldsymbol{\xi}_0(T_0) \in H$. We suppose also, without restriction or loss of generality that $\boldsymbol{\xi}(0,k) \in H$ which implies that $\mathbf{P}d\boldsymbol{\xi}(0,k) = d\boldsymbol{\xi}(0,k)$. However, in general:

$$\mathbf{P}d\boldsymbol{\xi}(T_0, k_0 + dk) \neq d\boldsymbol{\xi}(T_0, k_0 + dk)$$

but we have

$$\boldsymbol{\xi}(T(k_0 + dk), k_0 + dk) = \boldsymbol{\xi}(0, k_0 + dk) \in H$$

This can be expressed as $\mathbf{e}^T\left(\boldsymbol{\xi}(T_0 + dT, k_0 + dk) - \boldsymbol{\xi}_0(0)\right) = 0$ and with the first order approximation

$$\begin{aligned}
\boldsymbol{\xi}(T_0 + dT, k_0 + dk) - \boldsymbol{\xi}_0(0) &\approx \frac{\partial \boldsymbol{\xi}}{\partial t}(T_0, k_0)dT + \frac{\partial \boldsymbol{\xi}}{\partial k}(T_0, k_0)dk \\
&= \frac{\partial \boldsymbol{\xi}}{\partial t}(0, k_0)dT + \mathbf{M}(T_0)\frac{\partial \boldsymbol{\xi}}{\partial k}(0, k_0)dk + \mathbf{V}(T_0)dk
\end{aligned}$$

we have

$$\begin{aligned}
0 &\approx \mathbf{e}^T\left(\mathbf{e}\left\|\frac{\partial \boldsymbol{\xi}}{\partial t}(0, k_0)\right\| dT + \mathbf{M}(T_0)\frac{\partial \boldsymbol{\xi}}{\partial k}(0, k_0)dk + \mathbf{V}(T_0)dk\right) \\
dT &\approx -\frac{\mathbf{e}^T\left(\mathbf{M}(T_0)\frac{\partial \boldsymbol{\xi}}{\partial k}(0, k_0)dk + \mathbf{V}(T_0)dk\right)}{\left\|\frac{\partial \boldsymbol{\xi}}{\partial t}(0, k_0)\right\|}
\end{aligned}$$

therefore

$$\begin{aligned}
\frac{\partial \boldsymbol{\xi}}{\partial t}(T_0, k_0)dT &\approx -\mathbf{e}\mathbf{e}^T\left(\mathbf{M}(T_0)\frac{\partial \boldsymbol{\xi}}{\partial k}(0, k_0)dk + \mathbf{V}(T_0)dk\right) \\
\boldsymbol{\xi}(T_0 + dT, k_0 + dk) - \boldsymbol{\xi}_0(0) &\approx (\mathbf{I} - \mathbf{e}\mathbf{e}^T)\left(\mathbf{M}(T_0)\frac{\partial \boldsymbol{\xi}}{\partial k}(0, k_0)dk + \mathbf{V}(T_0)dk\right) \\
&= \mathbf{P}\left(\mathbf{M}(T_0)\frac{\partial \boldsymbol{\xi}}{\partial k}(0, k_0)dk + \mathbf{V}(T_0)dk\right)
\end{aligned}$$

And because of

$$\begin{aligned}
\boldsymbol{\xi}(T_0 + dT, k_0 + dk) - \boldsymbol{\xi}_0(0) &= \boldsymbol{\xi}(0, k_0 + dk) - \boldsymbol{\xi}_0(0) \\
&\approx \frac{\partial \boldsymbol{\xi}}{\partial k}(0, k_0)dk = \mathbf{P}\frac{\partial \boldsymbol{\xi}}{\partial k}(0, k_0)dk
\end{aligned}$$

We get

$$\begin{aligned}
\mathbf{P}\left(\mathbf{M}(T_0)\frac{\partial \boldsymbol{\xi}}{\partial k}(0, k_0)dk + \mathbf{V}(T_0)dk\right) &\approx \mathbf{P}\frac{\partial \boldsymbol{\xi}}{\partial k}(0, k_0)dk \\
\frac{\partial \boldsymbol{\xi}}{\partial k}(0, k_0)dk &= \left[\mathbf{I} - \mathbf{P}\mathbf{M}(T_0)\right]_H^{-1}\mathbf{P}\mathbf{V}(T_0)dk
\end{aligned}$$

Where $[\mathbf{A}]_H^{-1}$ is the inverse of the matrix $\mathbf{A}$ restricted to the hyperplane H.

If we use the last equation in the derivation of dT. we find

$$\frac{\partial T}{\partial k} \approx -\frac{\mathbf{e}^T\left(\mathbf{M}(T_0)[\mathbf{I} - \mathbf{P}\mathbf{M}(T_0)]_H^{-1}\mathbf{P}\mathbf{V}(T_0) + \mathbf{V}(T_0)\right)}{\left\|\frac{\partial \boldsymbol{\xi}}{\partial t}(0, k_0)\right\|}$$

Which can also be expressed with $\mathbf{k}$ being a vector with $\frac{d\mathbf{x}}{dt} = \mathbf{F}(\mathbf{x}(t), \mathbf{k})$ and $\mathbf{V}$ being a matrix as

$$\frac{\partial T}{\partial \mathbf{k}} \approx -\frac{\mathbf{e}^T \left(\mathbf{M}(T_0)[\mathbf{I} - \mathbf{P}\mathbf{M}(T_0)]_H^{-1}\mathbf{P}\mathbf{V}(T_0) + \mathbf{V}(T_0)\right)}{\left\| \frac{\partial \boldsymbol{\xi}}{\partial t}(0, \mathbf{k}_0) \right\|}$$

With the last equation, we can calculate our robustness quantifier ρ_S. We numerically integrate the variational equations for $\mathbf{M}(t)$ and $\mathbf{V}(t)$ over one period using MATLAB, which allows us to estimate $\frac{\partial T}{\partial \mathbf{k}}$. To increase the precision of this estimate, we start the integration at different points of the cycle and average the results. Finally, we use the following relation to calculate $\rho_S(\mathbf{k})$:

$$\frac{\partial T}{\partial \mathbf{k}} \frac{\mathbf{k}}{T} = \frac{\frac{\partial T}{T}}{\frac{\partial \mathbf{k}}{\mathbf{k}}} = \frac{\partial \log(T)}{\partial \log(\mathbf{k})}$$

A.4 Autocatalytic model

The autocatalytic model, as described in [3] contains 8 states variables and 7 reactions with 7 individual parameters (Figure 2A). In the equation system below square brackets denote concentrations, and names inscribed in these brackets denote (phospho)proteins or complexes thereof.

$$
\begin{aligned}
\frac{d[\mathrm{KaiA}]}{dt} &= \mathrm{k}_5[\mathrm{KaiABC}*] - \mathrm{k}_1[\mathrm{KaiA}][\mathrm{KaiC}] - \mathrm{k}_3[\mathrm{KaiAC}*][\mathrm{KaiA}][\mathrm{KaiC}] \\
\frac{d[\mathrm{KaiB}]}{dt} &= \mathrm{k}_6[\mathrm{KaiBC}*] - \mathrm{k}_4[\mathrm{KaiAC}*][\mathrm{KaiB}] \\
\frac{d[\mathrm{KaiC}]}{dt} &= \mathrm{k}_7[\mathrm{KaiC}*] - \mathrm{k}_1[\mathrm{KaiA}][\mathrm{KaiC}] - \mathrm{k}_3[\mathrm{KaiAC}*][\mathrm{KaiA}][\mathrm{KaiC}] \\
\frac{d[\mathrm{KaiC}*]}{dt} &= \mathrm{k}_6[\mathrm{KaiBC}*] - \mathrm{k}_7[\mathrm{KaiC}*] \\
\frac{d[\mathrm{KaiAC}]}{dt} &= \mathrm{k}_1[\mathrm{KaiA}][\mathrm{KaiC}] - \mathrm{k}_2[\mathrm{KaiAC}] \\
\frac{d[\mathrm{KaiAC}*]}{dt} &= \mathrm{k}_2[\mathrm{KaiAC}] + \mathrm{k}_3[\mathrm{KaiAC}*][\mathrm{KaiA}][\mathrm{KaiC}] - \mathrm{k}_4[\mathrm{KaiAC}*][\mathrm{KaiB}] \\
\frac{d[\mathrm{KaiBC}*]}{dt} &= \mathrm{k}_5[\mathrm{KaiABC}*] - \mathrm{k}_6[\mathrm{KaiBC}*] \\
\frac{d[\mathrm{KaiABC}*]}{dt} &= \mathrm{k}_4[\mathrm{KaiAC}*][\mathrm{KaiB}] - \mathrm{k}_5[\mathrm{KaiABC}*]
\end{aligned}
$$

To initialize our iterative exploration of parameter space, we use the following parameter vector reported in [3].

$$\mathbf{k} = [10^{-4}\ \mathrm{mol}^{-1}\mathrm{h}^{-1}, 0.40\ \mathrm{h}^{-1}, 0.45\ \mathrm{M}^{-2}\mathrm{h}^{-1}, 3.65\ \mathrm{h}^{-1}, 4.00\ \mathrm{h}^{-1}, 0.90\ \mathrm{h}^{-1}, 0.18\ \mathrm{h}^{-1}]$$

The total concentrations of the relevant molecules are $[\Sigma\mathrm{KaiA}] = 3.0\ \mu M$, $[\Sigma\mathrm{KaiB}] = 1.0\ \mu M$ and $[\Sigma\mathrm{KaiC}] = 3.5\ \mu M$.

A.5 Two phosphorylation sites model

The two-sites model contains 4 states variables and 8 reactions with 12 parameters [4] (Figure 2B). [KaiA] is expressed as a function of [KaiC^S]:

$$
\begin{aligned}
[\mathrm{KaiA}]\left([\mathrm{KaiC}^S]\right) &= [\mathrm{KaiA}] = \max\left\{0, [\Sigma\mathrm{KaiA}] - 2[\mathrm{KaiC}^S]\right\} \\
[\mathrm{KaiC}] &= [\Sigma\mathrm{KaiC}] - [\mathrm{KaiC}^T] - [\mathrm{KaiC}^{ST}] - [\mathrm{KaiC}^S] \\
\frac{d[\mathrm{KaiC}^T]}{dt} &= \frac{\mathrm{k}_4[\mathrm{KaiA}]}{\mathrm{k}_{12} + [\mathrm{KaiA}]}[\mathrm{KaiC}] + \frac{\mathrm{k}_9[\mathrm{KaiA}]}{\mathrm{k}_{12} + [\mathrm{KaiA}]}[\mathrm{KaiC}^{ST}] \\
&\quad - \left(\mathrm{k}_1 + \frac{\mathrm{k}_8[\mathrm{KaiA}]}{\mathrm{k}_{12} + [\mathrm{KaiA}]}\right)[\mathrm{KaiC}^T] - \frac{\mathrm{k}_5[\mathrm{KaiA}]}{\mathrm{k}_{12} + [\mathrm{KaiA}]}[\mathrm{KaiC}^T] \\
\frac{d[\mathrm{KaiC}^{ST}]}{dt} &= \frac{\mathrm{k}_5[\mathrm{KaiA}]}{\mathrm{k}_{12} + [\mathrm{KaiA}]}[\mathrm{KaiC}^T] + \frac{\mathrm{k}_6[\mathrm{KaiA}]}{\mathrm{k}_{12} + [\mathrm{KaiA}]}[\mathrm{KaiC}^S] \\
&\quad - \left(\mathrm{k}_2 + \frac{\mathrm{k}_{10}[\mathrm{KaiA}]}{\mathrm{k}_{12} + [\mathrm{KaiA}]}\right)[\mathrm{KaiC}^{ST}] - \frac{\mathrm{k}_9[\mathrm{KaiA}]}{\mathrm{k}_{12} + [\mathrm{KaiA}]}[\mathrm{KaiC}^{ST}] \\
\frac{d[\mathrm{KaiC}^T]}{dt} &= \left(\mathrm{k}_2 + \frac{\mathrm{k}_{10}[\mathrm{KaiA}]}{\mathrm{k}_{12} + [\mathrm{KaiA}]}\right)[\mathrm{KaiC}^{ST}] + \frac{\mathrm{k}_7[\mathrm{KaiA}]}{\mathrm{k}_{12} + [\mathrm{KaiA}]}[\mathrm{KaiC}] \\
&\quad - \left(\mathrm{k}_3 + \frac{\mathrm{k}_{11}[\mathrm{KaiA}]}{\mathrm{k}_{12} + [\mathrm{KaiA}]}\right)[\mathrm{KaiC}^S] - \frac{\mathrm{k}_6[\mathrm{KaiA}]}{\mathrm{k}_{12} + [\mathrm{KaiA}]}[\mathrm{KaiC}^S]
\end{aligned}
$$

To initialize our iterative exploration of parameter space, we use the following parameter vector reported in [4].

$$
\begin{aligned}
\mathbf{k} &= [0.21\ \mathrm{h}^{-1}, 0.31\ \mathrm{h}^{-1}, 0.11\ \mathrm{h}^{-1}, 0.4791\ \mathrm{h}^{-1}, 0.2129\ \mathrm{h}^{-1}, 0.5057\ \mathrm{h}^{-1}, \\
&\quad 0.0532\ \mathrm{h}^{-1}, 0.7985\ \mathrm{h}^{-1}, 0.173\ \mathrm{h}^{-1}, -0.3194\ \mathrm{h}^{-1}, -0.1331\ \mathrm{h}^{-1}, 0.43\ M]
\end{aligned}
$$

The total concentrations are $[\Sigma\mathrm{KaiA}] = 1.3\mu M$ and $[\Sigma\mathrm{KaiC}] = 3.4\mu M$.

A.6 Computational requirements

The behavior we chose is challenging to analyze, because it involves cyclic and not fixed point (steady-state) behavior. We thus need to integrate models over multiple periods to measure properties with any precision. To give but one example, to estimate period and amplitude with less than 0.1% of error for the two-sites model in the Monte Carlo integration requires on average 0.17 seconds for any one parameter vector on an Intel Xeon X5355 processor (2.66 GHz) using only one core. These numbers translate into 40 hours for the 9×10^5 vectors we sampled for the two-sites model.

For robustness quantification, finite parameter perturbations are the most costly, as they require 10^3 model integrations per parameter vector, resulting in 4-6 minutes of simulation time on one Xeon core. To estimate ρ_P for the 604 parameter vectors for the two-sites model requires approximately 30 hours. The next most costly procedure is estimation of robustness to molecular noise. For instance, to estimate ρ_N with less than one percent error requires more than one minute on seven cores of a dual quad-core Xeon X5355 processor, or 20 hours for all 604 sampled viable parameter vectors. Overall, estimation of all global and local robustness quantifiers for any one model require less than 5 days on a dual quad-core Xeon X5355 processor.

B Supplementary results for the cyanobacterial models

B.1 The region of viable parameters may be connected in parameter space.

We asked whether the region of viable parameters is connected in parameter space. For any high dimensional model whose governing equations that cannot be solved analytically, this is perhaps the most difficult problem in global analysis, for the following reason. The sample of viable parameters that our approach generates, albeit large, is finite, and comes from a multidimensional parameter space with uncountably many elements. In this parameter space, the set of all viable parameters may be connected, or, alternatively, it may be fragmented into many small islands of viable subsets. No sampling approach can prove which of these extremes (or a spectrum of possibilities in between) is the case. However, sampling can provide a hint as to which of these scenarios is closer to the truth. To this end we define a graph whose nodes are parameter vectors in the viable set, and where an edge connects two nodes if a straight line exists that preserves the oscillatory behavior for all points along the line. To determine whether two parameter vectors, say $\underline{\mathbf{k}}$ and $\overline{\mathbf{k}}$, are neighbors in this graph, we sampled a convex combination $\mathbf{k}_i = (1 - \frac{i}{M})\underline{\mathbf{k}} + \frac{i}{M}\overline{\mathbf{k}}$, using a finite sample M of points between $\underline{\mathbf{k}}$ and $\overline{\mathbf{k}}$. We assessed whether all of these M points preserved proper oscillatory behavior, which suggests that the straight line connecting $\underline{\mathbf{k}}$ and $\overline{\mathbf{k}}$ lies in its entirety in the viable set. Figure S1 shows a projection of the structure of the entire graph into the two-dimensional space defined by parameters k_5 and k_6 for the autocatalytic model, and into the two-dimensional space defined by parameters k_1 and k_2 for the two-sites model.

For both models, the graph thus estimated consists of one connected component comprising the vast majority of parameter vectors, and few isolated nodes. Specifically, in the autocatalytic model, only 0.7% (12 of 1828) parameter vectors are not in this connected component. In the two-sites model 1.3% (8 out of 604) parameter vectors are not in this component. The isolated parameter vectors lie close to the boundary of the viable parameter volume. For these analyses, we used $M = 10 \cdot \|\underline{\mathbf{k}} - \overline{\mathbf{k}}\|$ resulting in a sample of 10 points per order of magnitude change along each straight line connecting any two parameter vectors, corresponding to a 25% difference in parameter values between two successive sampling points. Increasing the density of the sampling did not affect these results qualitatively (not shown). The parameter pairs in Figure S1 were chosen for the projection, because they best illustrate that the set of viable parameter vectors is not convex.

B.2 Robustness to temperature changes.

We now address in more details the question of temperature compensation for the studied models. Ideally, to estimate an oscillator's ability to compensate for temperature changes the Arrhenius equation, which predicts the effect of temperature on chemical reaction rates [3,14], would be used. However, this approach requires knowledge of the activation energy for each reaction in a circuit, which is usually not available. Still, the sensitivity of the period to such parameter changes [3, 14, 15] can be studied. Specifically, the values $\alpha_i = \partial \log(T)/\partial \log(k_i)$ can be calculated using our derivation in SI A.3.4. In general a faster reaction decreases the period of the cycle ($\alpha_i < 0$). In order to have temperature compensation in any cycle, at least one of the values α_i should be positive [3, 14]. For the autocatalytic model, α_4 is positive and for the two-sites model $\alpha_3, \alpha_6, \alpha_9, \alpha_{10}$ are positive, therefore temperature compensation is possible in both models. To quantify this aspect of robustness with finite perturbations, we simply assume that an increase, or a decrease, of the temperature increases, respectively decreases, all circuit parameters with independent factors. To estimate the robustness value, we used the same approach as ρ_P as described in the main text of the article.

B.3 KaiAC complex formation and robustness in the autocatalytic model.

Figure S2C plots, for the autocatalytic model, ρ_N against the model parameter k_1 that is most highly correlated with it (Spearman r = 0.921, p $< 10^{-323}$, n = 1828). The Figure shows that $\rho_N < 0.5$ for more than half the range of viable parameters. All other parameters show a partial Spearman rank correlation with ρ_N (controlling for k_1) lower than $r < 0.35$. Parameter k_1 governs the rate at which KaiAC forms from KaiA and KaiC. Its importance can be explained by the disproportionate effect a decrease in k_1 has when k_1 is already low. For example, when k_1 is equal to 4×10^{-5}, this complex forms at an average rate of 0.76h^{-1}. If k_1 decreases modestly to 10^{-5}, this rate decreases to 0.19h^{-1} or one complex formation every five hours. Because this reaction starts the cycle, the fluctuations in its rate can spread and strongly affect the period.

B.4 KaiC^S and robustness in the two-sites model.

In the two-sites model, the parameters most highly correlated with ρ_N are k_2 and k_3 (Spearman r = 0.629 and r = 0.414, respectively, p $< 10^{-323}$, n = 604). All other parameters show a partial Spearman rank correlation with ρ_N (controlling for k_2 and k_3) lower than r $<$ 0.12. Figure S2D shows a scatterplot of ρ_N against model parameter k_2 over the entire range of viable parameters that spans four orders of magnitude. With a few exceptions, ρ_N is smaller than 0.5 only for one quarter of this range. Parameter k_2 and k_3 represent the rates of the dephosphorylation reactions KaiCST →KaiC^S and KaiC^S →KaiC, respectively. KaiC^S is a critical element of the negative feedback loop (red bar in Figure 2B) that inhibits the action of KaiA. This observation explains the importance of the reactions that form and destroy KaiC^S. Low values of k_2 combined with finite numbers of reacting molecules lead to greater noise in the formation of KaiC^S from KaiCST. Its concentration will thus fluctuate to a greater extent, and these fluctuations can then be further amplified by feedback.

B.5 The distribution of local robustness in the viable region of parameter space.

A remaining question regards the relationship between robustness quantifiers $\boldsymbol{\rho}$ and the center of the set of viable parameters. Naively, one might assume that robustness might be highest in this center, and that the value of any robustness quantifier decreases from this center. However, this is not generally the case (Figure S6A-E). Specifically, only 3 and 2 out of our five robustness quantifiers show this expected distance dependency for the autocatalytic and two-sites model, respectively, and none of these associations exceed r = 0.25. Average local robustness ρ_T is also not higher at the center (Figure S6F). The same holds if ρ_T is calculated through multiplication rather than averaging (not shown). In addition, regions of parameter space exist that have higher average robustness ρ_T (Figure 5) than the center, and there is a negative association between a parameter vector's ρ_T and its distance to the parameter vector with the highest ρ_T. This association is higher for the autocatalytic model (Spearman r = −0.355; p $< 10^{-323}$, n = 1828) than for the two-sites model (r = −0.196; p = 1.15×10^{-6}, n = 604). These observations further underscore the higher robustness of the two-sites model. They show that in the two-sites model, robustness decreases more slowly as the distance from the region of highest robustness increases. Figure S7 illustrates this relationship for a two-dimensional projection of parameter space.

C Robustness analysis of the Goodwin model

The aim of this analysis is to show the feasibility of the method on models that differ in their structure from the cyanobacterial models. We choose to analyze the Goodwin model for its generality, as most of the eukaryotic circadian models are based on it [16, 17]. Our application of the glocal method to this model comprises the global analysis and two local analyses: robustness to molecular noise ρ_N and the cycle attraction ρ_A.

C.1 Model and equations

We used a three-variable model, based on the Goodwin oscillator [16–18], a generic model of circadian oscillators. We took the same equations for this model as used by Gonze et al. in [18]. The first component X may stand for mRNA [17]. The protein Y is produced from the mRNA and is activating the protein Z. X is inhibited by Z. All components are degraded following Michaelis-Menten kinetics [18]. The starting point of our PCA expansion is the parameter vector published in [18]. All parameters are allowed to fluctuate over three orders of magnitude in the range [0.01, 10] except the Hill coefficient, which is maintained constant at $n = 4$. The equations of the three state variables contain ten parameters:

$$\begin{aligned}
\frac{dX}{dt} &= k_1 \frac{k_2^4}{k_2^4 + Z^4} - k_3 \frac{X}{k_4 + X} \\
\frac{dY}{dt} &= k_5 X - k_6 \frac{Y}{k_7 + Y} \\
\frac{dZ}{dt} &= k_8 Y - k_9 \frac{Z}{k_{10} + Z}
\end{aligned}$$

C.2 Results of the global analysis

For the global analysis, we defined the following systemic properties for the Goodwin model. First, the period has to be in the range of [21.6, 26.4] hours which represents a 10% variation around the value of 24 hours. The relative amplitude of the oscillations of the output (that we choose to be the second component, Y) has to be at least 40% of the peak value. Finally, the output component should have a peak value in the ±10% range around the nominal value which is 0.4.

Using these criteria, we performed three global analyses in parallel and the results are very similar, with a normalized volume R of 1.037, 1.039 and 1.036 for 10^5 parameter vectors tested during Monte Carlo integration. Analyzing the principal components of the set of viable parameter vectors shows that the parameters controlling the concentration of Z (k_8 and k_9) are strongly correlated (Pearson's r = 0.922, p = $2.14 \cdot 10^{-287}$, Figure S9B): the most constrained direction (standard deviation of 0.0717, lower arrow in S9A) has an angle of 23 degrees with the vector $\mathbf{k}_{min} = (0, 0, 0, 0, 0, 0, 0, 1, -1, 0)$. At the other extreme, the direction $\mathbf{k}_{max} \approxeq (0, 1, 0, 0, 0, 0, 0, 1, 1, 1)$ shows a standard deviation of 1.17, which is the largest (upper arrow in S9A). These two results show the importance of a precise regulation of the feedback component Z: k_8 controls its production and k_9 its degradation. The other two parameters of the least constrained axis $\mathbf{k}_{max}$ are related to the concentration of Z (k_2, the inhibitory constant in the production of X, and k_{10}, the Michaelis constants for the degradation of Z). This freedom is due to the lack of direct constraints on the component Z: because Z has an effect on the systemic properties only through its feedback, a rescaling of the parameters k_2, k_8, k_9 and k_{10} in $\mathbf{k}_{max}$ would not affect the viability of the parameter vector.

C.3 Results of the local analysis

In order to study the impact of the parameters on specific robustness properties, we performed local analyses on all viable parameter vectors found with the global analysis. The first property we study is the robustness to molecular noise, using the measure defined in section A.3.2. The values of ρ_N span almost the whole possible range from 0.04 to 1 (Fig. S10A). The parameters most correlated with ρ_N are k_2, a constant in the production of X, and k_5, the production rate of Y, with a Spearman's rank correlation coefficient of r = 0.501 and r = −0.431, respectively (both have a p-value below 10^{-323}) as shown in Figure S10B.

The second robustness quantifier we applied to this model is ρ_A, which measures the attraction of the cycle (section A.3.3). It reflects how fast a small perturbation of the trajectory will be absorbed. Its values also range from 0 to 1 with a large fraction of the parameters vectors showing high robustness (Fig. S11A). The parameters most correlated with ρ_S are k_1, the production rate of X, and k_7, the Michaelis constant for the degradation of Y, with a Spearman's rank correlation coefficient of $r = 0.236$ ($p = 5.33 \cdot 10^{-15}$) and $r = -0.440$ ($p < 10^{-323}$),respectively, as shown in Figure S11B.

In conclusion, this analysis shows that our method is also applicable to models with a structure that differs from the models of the cyanobacterial circadian clock. Our results show the importance of the control of the feedback component for the systemic properties. The local analyses reflect the high robustness of this generic model with a large fraction of parameter vectors with values of 1 for their local robustness. We also find the region in parameter space with the highest robustness according to the local quantifiers.

References

1. Hengl S, Kreutz C, Timmer T Maiwald (2007) Data-based identifiability analysis of non-linear dynamical models. BMC Bioinformatics 23: 2612–2618.

2. Ljung L, Glad T (1994) On global identifiability for arbitrary model parametrizations. Automatica 30: 265–276.

3. Mehra A, Hong CI, Shi M, Loros JJ, Dunlap JC, et al. (2006) Circadian rhythmicity by autocatalysis. PLoS Comput Biol 2: 816-823.

4. Rust MJ, Markson JS, Lane WS, Fisher DS, O’Shea EK (2007) Ordered phosphorylation governs oscillation of a three-protein circadian clock. Science 318: 809-812.

5. Jaulin L, Kieffer M, Didrit O, Walter E (2001) Applied Interval Analysis. London: Springer.

6. Kuipers BJ (1994) Qualitative Reasoning: Modeling and Simulation with Incomplete Knowledge. Cambridge, MA: MIT Press.

7. Ackermann J (2002) Robust Control, The Parameter Space Approach. London: Springer.

8. Pikovsky A, Maistrenko Y (2003) Sychronization: Theory and Application, volume 109 of *II. Mathematics, Physics and Chemistry*. Dordrecht: Kulwer Academics Publishers.

9. Rosenblum MG, Pikovsky AS, Kurths J (1996) Phase synchronization of chaotic oscillators. Phys Rev Lett 76: 1804-1807.

10. Gillespie DT (1977) Exact stochastic simulation of coupled chemical reactions. J Phys Chem 81: 2340-2361.

11. Kageyama H, Nishiwaki T, Nakajima M, Iwasaki H, Oyama T, et al. (2006) Cyanobacterial circadian pacemaker: Kai protein complex dynamics in the KaiC phosphorylation cycle in vitro. Mol Cell 23: 161-171.

12. Goldbeter A (1996) Biochemical Oscillations and Cellular Rhythms. Cambridge: Cambridge University Press.

13. Khalil HK (1996) Nonlinear systems. Upper Saddle River: Prentice-Hall.

14. Ruoff P (1992) Introducing temperature-compensation in any reaction kinetic oscillator model. J interdiscipl Cycle Res 23: 92-99.

15. Zak DE, Stelling J, Doyle FJ III (2005) Sensitivity analysis of oscillatory (bio)chemical systems. Comput Chem Eng 29: 663-673.

16. Goodwin BC (1965) Oscillatory behavior in enzymatic control processes. Adv Enzyme Regul 3: 425-438.

17. Ruoff P, Rensing L (1996) The temperature-compensated Goodwin model simulates many circadian clock properties. J theor Biol 179: 275-285.

18. Gonze D, Bernard S, Waltermann C, Kramer A, Herzel H (2005) Spontaneous synchronization of coupled circadian oscillators. Biophys J 89: 120-129.

Supplementary figures

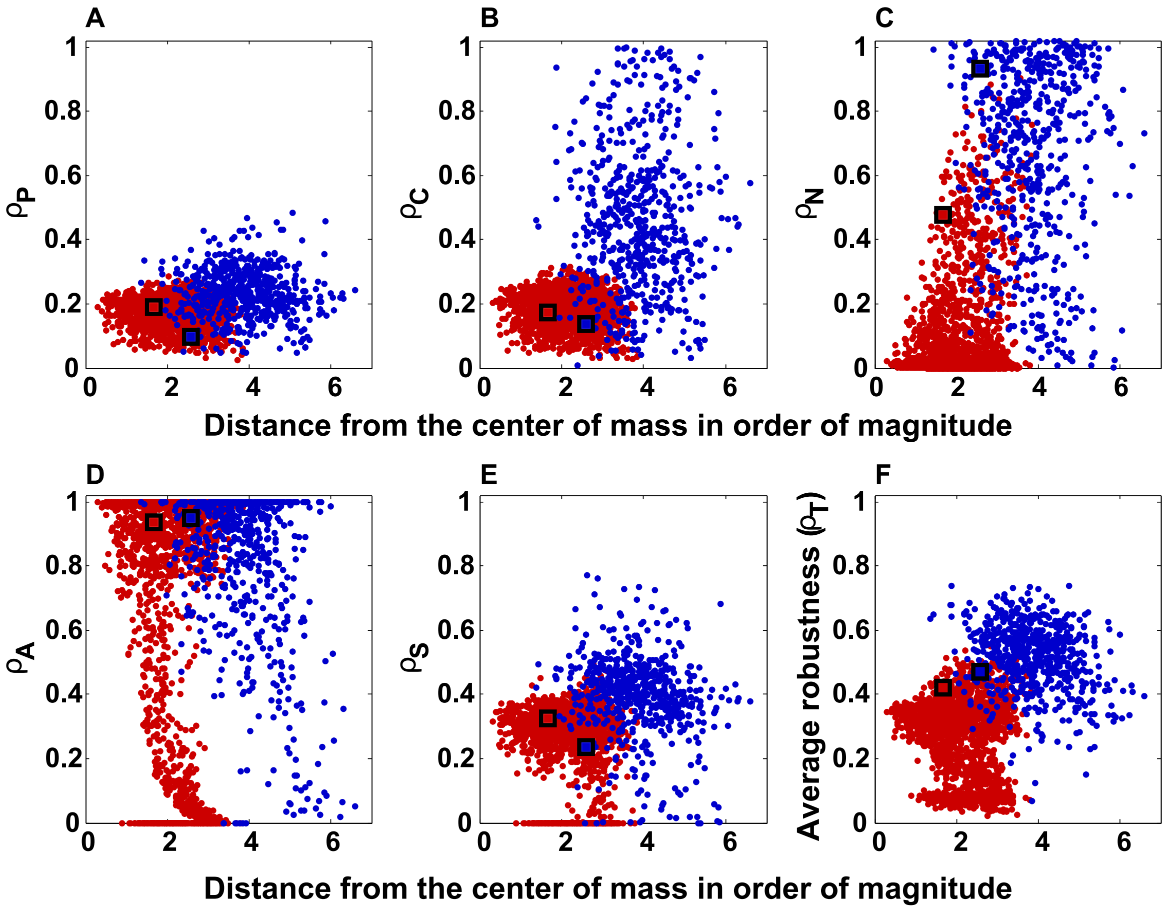

Figure S6: Local robustness is not maximal in the center of the viable parameter set. Shown are local robustness quantifiers (vertical axes) plotted against the distance of viable parameter vectors (horizontal axes) from the center of mass of the entire set of viable parameter vectors, for the autocatalytic model (red) and the two-sites model (blue): (A) robustness ρ_P to parameter perturbations, (B) robustness ρ_C to total concentration perturbations, (C) robustness ρ_N to molecular noise, (D) attraction of the cycle ρ_A, (E) sensitivity of the period ρ_S, (F) total robustness, ρ_T, defined as the arithmetic mean over all five robustness quantifiers. Maxima for all local robustness quantifiers are not found near the center of mass, and there is only a weak correlation between a parameter vectors distance from this center and local robustness. The two squares in each panel correspond to previously published parameter vectors for each model [3, 4]. The sparsity of data points near the center of mass stems from the fact that in a high-dimensional ball or ellipsoid, most of the mass is concentrated near its surface and not near its center.

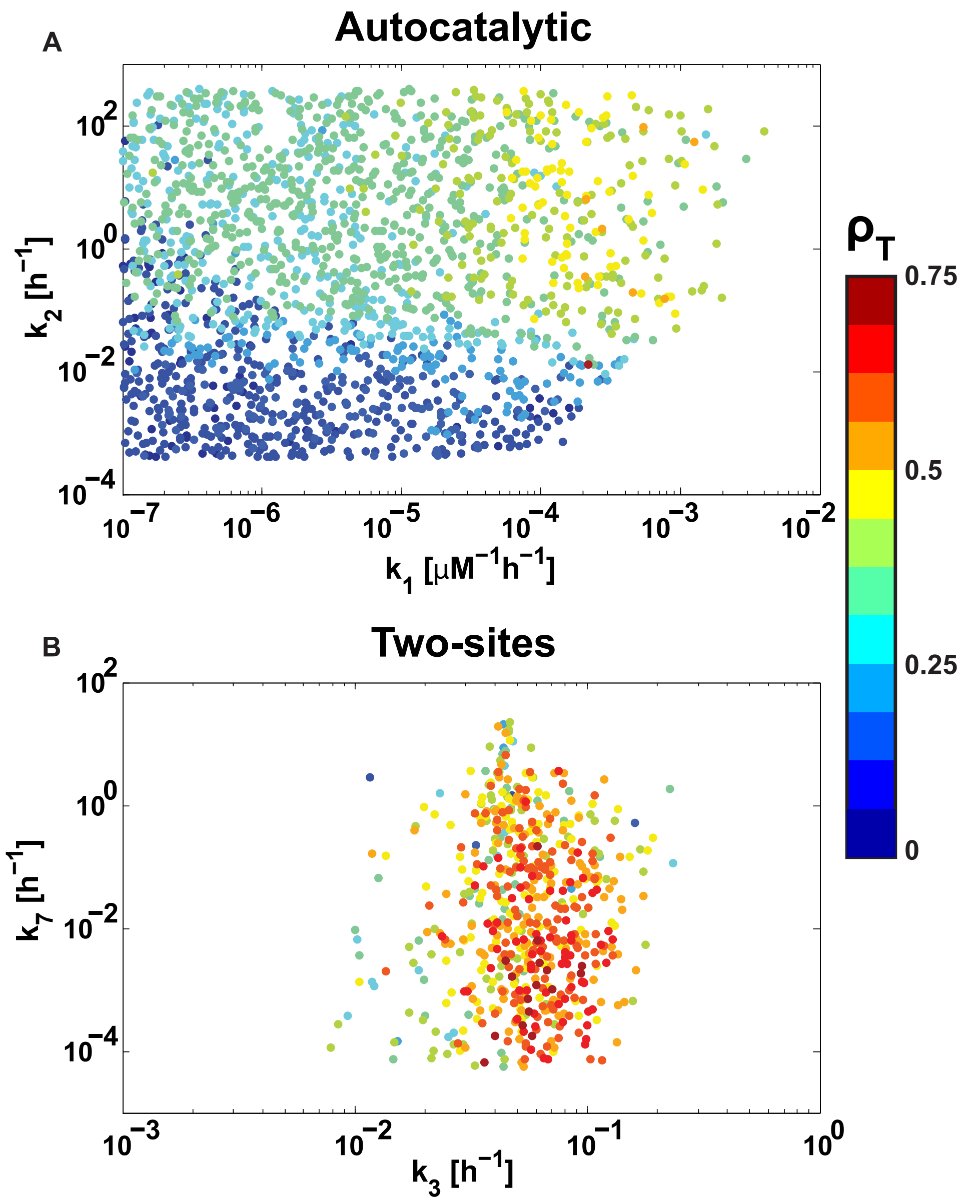

Figure S7: Local robustness is much greater over wide regions of parameter space for the two-sites model. Color codes indicate average local robustness ρ_T for the autocatalytic model (A) and the two-sites model (B). ρ_T is shown in a two-dimensional projection of parameter space onto parameters k_1 and k_2 in (A), as well as k_3 and k_7 in (B), which are the parameters most highly correlated with ρ_T. Color coding ranges from blue ($\rho_T = 0$) to red ($\rho_T = 0.737$, the maximum observed between both models).

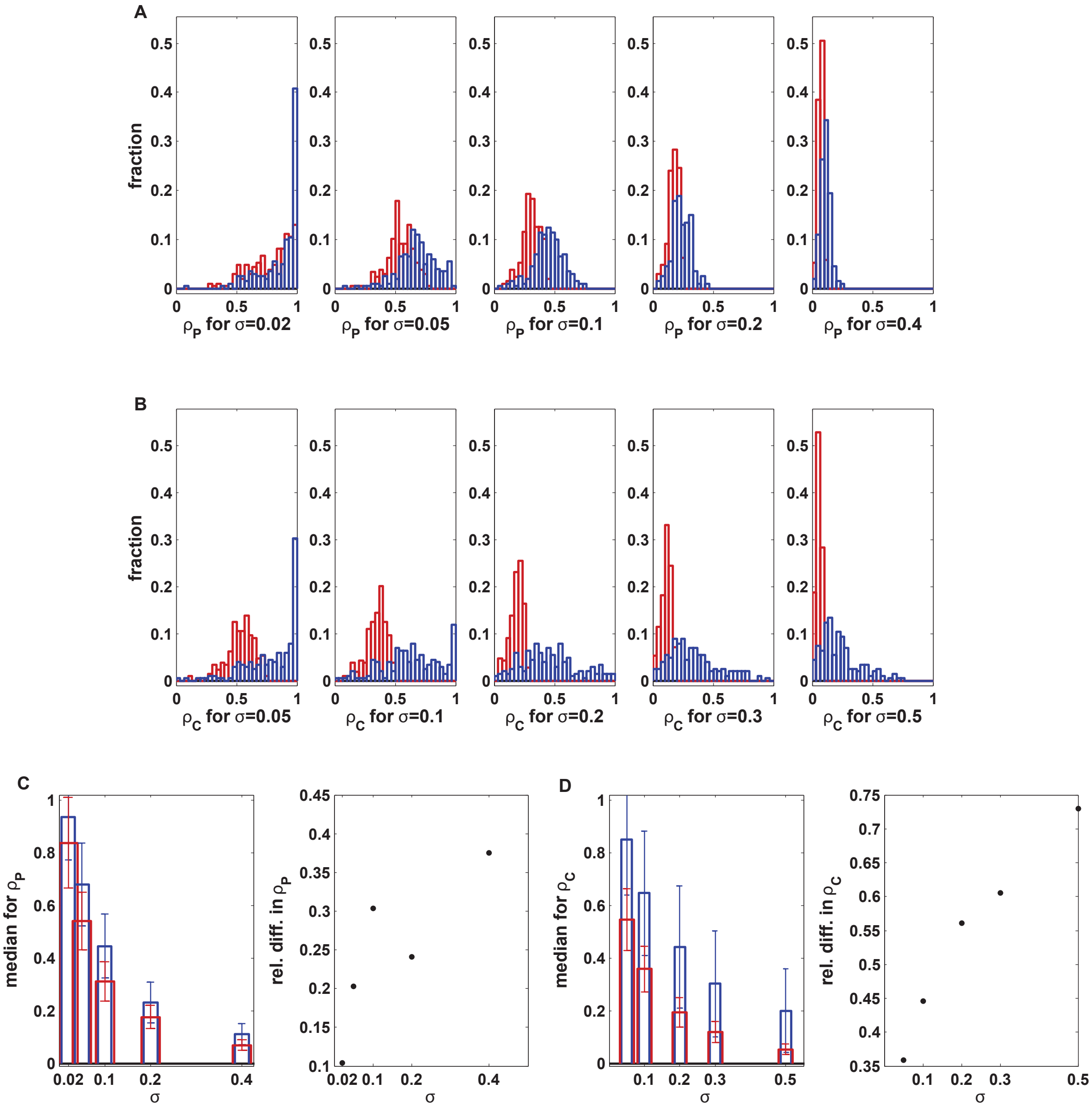

Figure S8: Distribution of local robustness to (A) parameter perturbations ρ_P and (B) total concentration perturbations ρ_C for different values of the standard deviation σ of the respective perturbations; autocatalytic model in red; two-sites model in blue. (C) and (D) show the median values of ρ_P and ρ_C, respectively, for both models (left panels) and the relative difference between the medians (right panels) as a function of σ. Based on this analysis, we choose $\sigma = 0.2$ for assessing local robustness throughout the remainder of the manuscript, because this value of σ yields a broad distribution of ρ_P and ρ_C and good discrimination between the two models.

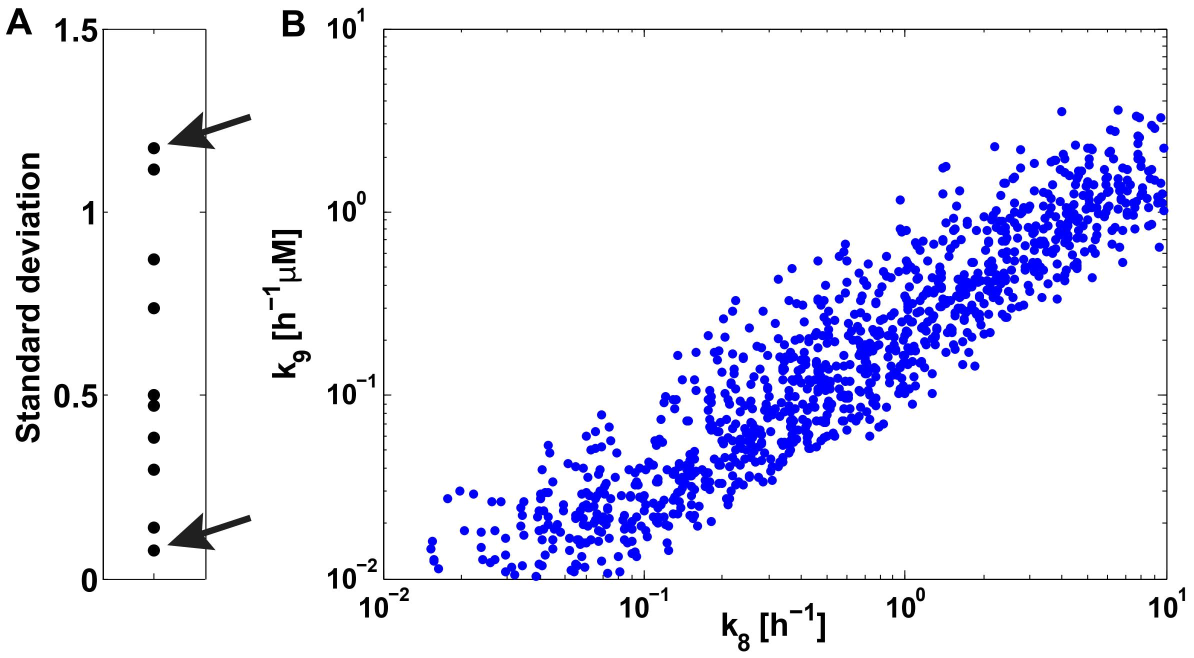

Figure S9: Results of the global analysis for the Goodwin model. (A) Distribution of the standard deviations along the principal axes of the set of viable parameter vectors. (B) Projection of the viable parameter vectors in the plane (k_8, k_9) for the Goodwin model. Parameters k_8 and k_9 are, respectively, the production and the degradation rate of Z. Both parameters are strongly correlated, resulting in the axis ($\mathbf{k}_{min}$, see SI, section C) with the lowest standard deviation for the Goodwin model (lower arrow in A). On the opposite, the other direction, $\mathbf{k}_{max}$, shows a large allowable variation (upper arrow in A).

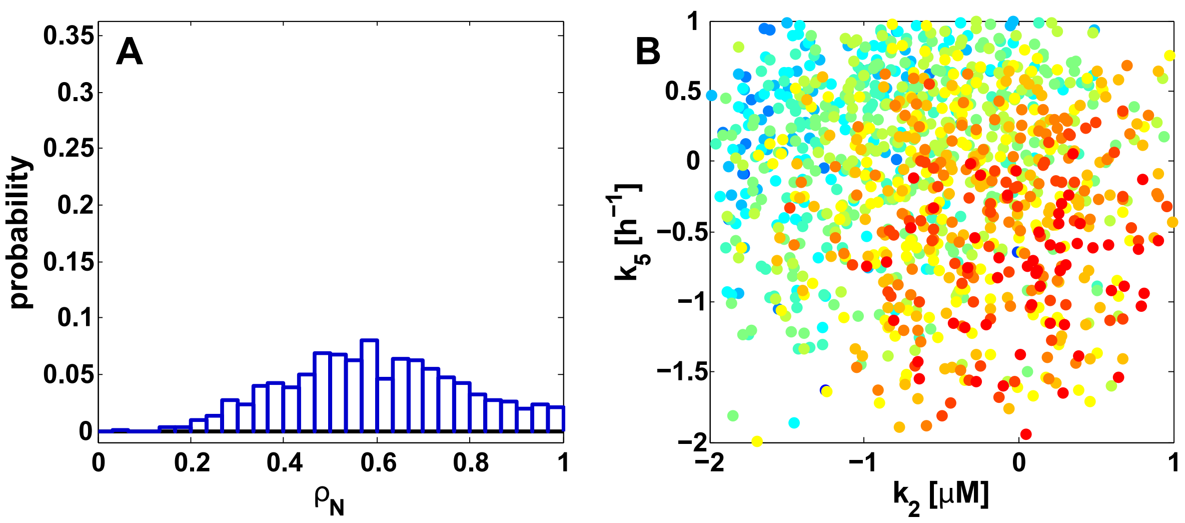

Figure S10: Robustness to molecular noise ρ_N for the Goodwin model. (A) Distribution of the values ρ_N. (B) Projection of the viable parameter vectors on the plane (k_2,k_5): colors correspond to the robustness value ranging from blue for a value of 0 to red for 1. Moreover, k_2 is a constant in the production of X, and k_5 is the production rate of Y.

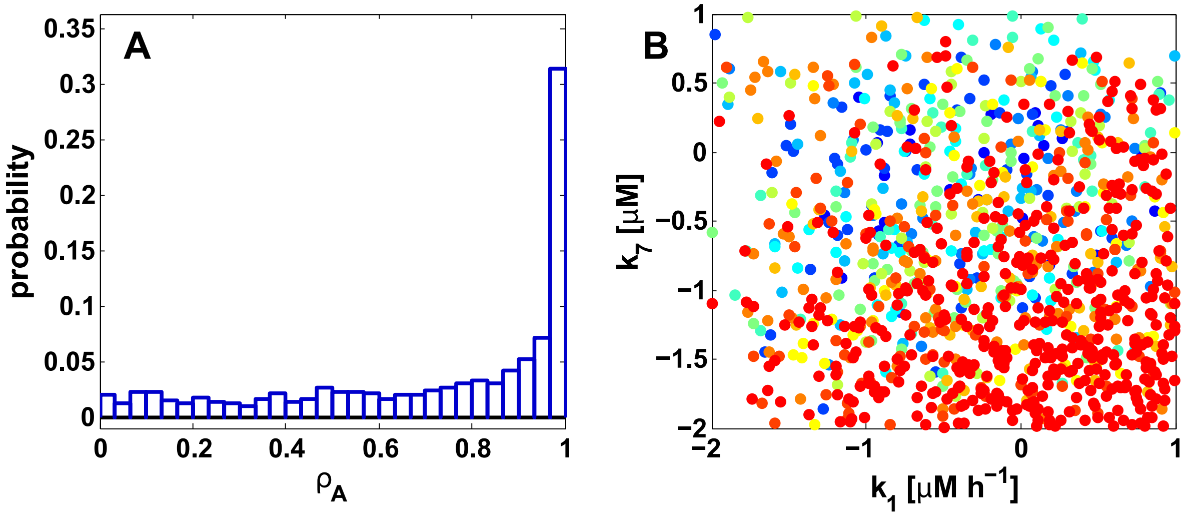

Figure S11: Cycle attraction ρ_A for the Goodwin model. (A) Distribution of the values ρ_A. (B) Projection of the viable parameter vectors on the plane (k_7,k_8), colors correspond to the robustness value ranging from blue for a value of 0 to red for 1. Moreover, k_1 is the production rate of X, and k_7 is the Michaelis constant for the degradation of Y.
